# Structural Characterization and Hypoglycemic Mechanism of Pumpkin Seed Polysaccharides

**DOI:** 10.3390/foods15213788

**Published:** 2026-10-22

**Authors:** Junyun Luo, Yuxiao Wu, Shu Cai, Jiaying Li, Yili Li, Aimei Zhou

**Affiliations:** Guangdong Provincial Key Laboratory of Nutraceuticals and Functional Foods, College of Food Science, South China Agricultural University, Guangzhou 510642, China; 18383555886@163.com (J.L.);

**Keywords:** pumpkin seed polysaccharides, structural identification, hypoglycemic

## Abstract

This study investigated three pumpkin seed polysaccharide fractions (PSP-1-1, PSP-1-2, and PSP-1-3) with molecular weights of 29,797 Da, 11,209 Da, and 4346 Da, respectively, all containing both α- and β-glycosidic linkages. Structural analysis revealed arabinose, galactose, and glucose as predominant residues in PSP-1-1, while PSP-1-2 and PSP-1-3 additionally contained xylose, with all fractions sharing common structural units. All three fractions were associated with dose-dependent increases in GLP-1 secretion from L cells and with upregulation of genes in the sweet taste receptor pathway. The pooled fraction PSP-1s showed higher gene expression than individual fractions and was therefore selected for in vivo studies. In diabetic mice, PSP-1s intervention alleviated weight loss, reduced hyperphagia and polydipsia, lowered blood glucose, improved glucose tolerance and dyslipidemia, and decreased GSP, insulin, and HOMA-IR while increasing QUICKI. Qualitative histological observations suggested less pronounced pancreatic islet abnormalities following PSP-1s intervention. PSP-1s increased serum GLP-1 concentrations and colonic SCFA concentrations and altered gut microbiota composition. Collectively, the hypoglycemic effects of PSP-1s were accompanied by changes in serum GLP-1, colonic SCFAs, gut microbiota composition, and hypothalamic GLP-1R and appetite-related gene expression. Together with the cellular findings, these observations suggest potential mechanisms involving sweet taste receptor-related signaling and microbiota-associated GLP-1 regulation. However, pathway activation and the causal contribution of gut–brain signaling remain to be established. These findings support further investigation of PSP-1s as a functional food ingredient with potential glucose-regulating activity.

## 1. Introduction

The development of bioactive compounds derived from natural products as supplements or alternatives to pharmaceuticals for diabetes management has emerged as a significant trend in the field of functional food development. Among these compounds, polysaccharides are regarded as having substantial potential for applications in diabetes regulation. Currently, numerous natural polysaccharides have been found to exhibit favorable hypoglycemic effects, such as polysaccharides from Finger citron [1], Coix seed [2], *Ganoderma lucidum* [3], and *Polygonatum cyrtonema* Hua [4].

Pumpkin seeds, derived as a primary by-product from the processing of pumpkin (*Cucurbita moschata* (Duchesne ex Lam.) Duchesne ex Poir.), are recognized for their edible and medicinal attributes. They are abundant in various nutritive and functional constituents, including oil, proteins, phenols, flavonoids, and polysaccharides [5,6]. Pumpkin seed oil is the main product from processing pumpkin seeds, but the residue left after oil extraction remains underutilized. Notably, polysaccharides constitute one of the effective functional components within this oil-extracted pumpkin seed residue, yet research on pumpkin seed polysaccharides remains limited both domestically and internationally. Recently, our research group isolated and purified three previously unreported pumpkin seed polysaccharides (PSP-1-1, PSP-1-2, and PSP-1-3) from pumpkin seed meal, all of which exhibited good in vitro hypoglycemic activity [5]. Monosaccharide composition analysis revealed a high degree of structural homology among them, as they were all composed of galactose, arabinose, glucose, and xylose. Specifically, the relative molar ratios of these four monosaccharides in PSP-1-1, PSP-1-2, and PSP-1-3 were 61.5:24.4:5.9:0.5, 38.2:14.8:28.6:8.9, and 38.8:14.2:31.7:7.3, respectively.

Polysaccharides, as a class of biologically active macromolecules, have difficulty in penetrating the intestinal mucosal barrier to enter the bloodstream and reach target organs. Consequently, their biological effects are predominantly manifested within the gut [7]. Nevertheless, the precise mechanisms through which polysaccharides modulate glucose metabolism and regulate appetite remain inadequately understood. Current research suggests that the gut–brain axis is instrumental in the transmission of hypoglycemic signaling molecules [8]. The gut microbiota and the human host exist in a dynamic equilibrium characterized by mutual dependence and constraint, forming a symbiotic system. Furthermore, the gut maintains intricate connections with the brain, relaying various signals via the vagus nerve, spinal neurons, and other pathways. These signals, which may include gut hormones or cytokines secreted by immune cells, constitute the gut–brain axis through their interactions between the digestive tract and the central nervous system, thereby playing a role in and influencing the body’s energy metabolism.

In the gut–brain axis, glucagon-like peptide-1 (GLP-1), secreted by enteroendocrine L cells, serves as a crucial signaling molecule through which polysaccharides regulate glucose metabolism [9]. Its receptors are widely distributed in the brain, gastrointestinal tract, and pancreas, where they stimulate insulin secretion, enhance satiety, slow gastric emptying, and promote pancreatic islet cell regeneration, making GLP-1 a key therapeutic target for type 2 diabetes. There are two pathways by which polysaccharides promote GLP-1 secretion from L cells: First, the fermentation of polysaccharides by gut microbiota produces short-chain fatty acids (SCFAs), which in turn stimulate L cells to secrete GLP-1 [10]. For instance, polysaccharides from Shiitake [11], *Ganoderma lucidum* [3], White hyacinth bean [12], *Achyranthes bidentata* [13], and *Lycium barbarum* [14] have been reported to modulate the composition and abundance of gut microbiota, particularly by increasing bacteria that produce SCFAs. The elevation of SCFAs induces the release of more downstream target signaling molecule GLP-1, thereby regulating gut–brain axis signaling, suppressing appetite [15], and exerting hypoglycemic effects. Second, research has demonstrated that sweet taste receptors located on L cells play a role in the signaling pathway by which monosaccharides, polysaccharides, oligosaccharides, or carbohydrates stimulate GLP-1 secretion. Polysaccharides can enhance GLP-1 synthesis via sweet taste receptor-mediated pathways [4,16]. These findings provide a rationale for investigating whether the hypoglycemic effects of pumpkin seed polysaccharides are accompanied by changes in GLP-1 secretion, sweet taste receptor-related gene expression, and microbiota-associated metabolites.

Building upon the preceding analysis, this study initially characterized the structural properties of pumpkin seed polysaccharide fractions PSP-1-1, PSP-1-2, and PSP-1-3 through molecular weight determination, Fourier-transform infrared spectroscopy (FT-IR), and methylation analysis. Subsequently, utilizing the intestinal endocrine L-cell model NCI-H716 as the focal point of investigation, the study examined the effects of pumpkin seed polysaccharides on GLP-1 secretion and the expression of genes associated with the sweet taste receptor signaling pathway. Furthermore, animal experiments were conducted to assess the hypoglycemic activity of pumpkin seed polysaccharides. To explore potential mechanisms associated with these effects from the perspective of hypoglycemic signal transduction, serum GLP-1 levels, gut microbiota composition, colonic SCFA levels, hypothalamic GLP-1 receptor (GLP-1R) protein expression, and appetite-related gene expression were examined.

## 2. Materials and Methods

### 2.1. Chemicals and Materials

Pumpkin seeds used in this study were sourced from Xinjiang Aiyouwei Feed Food Co., Ltd. (Urumqi, China). The NCI-H716 cell line was purchased from the American Type Culture Collection (ATCC, Manassas, VA, USA). Gibco (Grand Island, New York, NY, USA) supplied Roswell Park Memorial Institute (RPMI) 1640 medium and fetal bovine serum (FBS). Methyl thiazolyl tetrazolium (MTT) was provided by Sigma-Aldrich (St Louis, MO, USA). The RC112, R223, and Q711 kits were procured from Nanjing Vazyme Biotech Co., Ltd. (Nanjing, China). Male C57BL/6N mice (NO. SCXK (ZHE) 2024-0011) were acquired from Beijing Vital River Laboratory Animal Technology Co., Ltd. (Beijing, China). The standard diet (PD1001) and high-fat diet (PD12493) were provided by Shuyishuer Biotechnology Co., Ltd. (Changzhou, China). Streptozotocin (STZ) and metformin were sourced from Shanghai Yuanye Bio-Technology Co., Ltd. (Shanghai, China). 3% isoflurane and hematoxylin-eosin (HE) staining solution were obtained from Solarbio (Beijing, China). Nanjing Jiancheng Bioengineering Institute (Nanjing, China) provided commercial kits for the measurement of TC (A111-1-1), TG (A110-1-1), LDL-C (A113-1-1), HDL-C (A112-1-1), glycated serum protein (GSP) (A037-2-1), insulin (H203-1-2), and biologically active GLP-1 (H294-1-2). Standards of acetic acid, propionic acid, butyric acid, isobutyric acid, valeric acid, and isovaleric acid were purchased from Sigma–Aldrich (Milan, Italy). Radioimmunoprecipitation assay (RIPA) lysis buffer, 10% (*w*/*v*) sodium dodecyl sulfate (SDS) solution, and polyvinylidene difluoride (PVDF) membranes were purchased from Merck KGaA (Darmstadt, Germany). Proteintech Group, Inc. (Chicago, IL, USA) supplied mouse monoclonal antibodies for β-actin (26196-1-AP, 1:500) and GLP-1R (66009-1-Ig, 1:5000), together with horseradish peroxidase (HRP)-conjugated goat anti-mouse secondary antibody (AWS0002, 1:5000). An enhanced chemiluminescence (ECL) kit was obtained from Thermo Fisher Scientific (Carlsbad, CA, USA). All remaining reagents used in this work were of analytical grade.

### 2.2. Preparation of Pumpkin Seed Polysaccharides

The isolation and purification of pumpkin seed polysaccharides were carried out based on our previous study [5]. In brief, the crude polysaccharide from pumpkin seeds was obtained through continuous phase-transition extraction and deproteinized using the Sevag method and papain. The resulting crude polysaccharide was dissolved in deionized water to a concentration of 15 mg/mL. After filtration through a 0.45 μm membrane, the solution was loaded onto a DEAE-52 cellulose column and eluted with water and NaCl solutions of varying concentrations (0.1, 0.3, 0.5, and 0.7 mol/L) in a gradient manner. The eluates were collected using an automated fraction collector, and their absorbance was determined by the phenol-sulfuric acid method, yielding a single symmetrical elution peak, which was designated as PSP-1 (Figure S1A). PSP-1 was further separated on a Sephadex-75 gel column, and three elution peaks were observed after water elution, which were named PSP-1-1, PSP-1-2, and PSP-1-3, respectively (Figure S1B). Additionally, the three polysaccharide fractions (PSP-1-1, PSP-1-2, and PSP-1-3) obtained after purification of PSP-1 through a Sephadex-75 gel column were pooled and designated as PSP-1s. Table S1 presents the physicochemical properties and yields of each pumpkin seed polysaccharide fraction.

### 2.3. Molecular Weight Detection

The molecular weights of PSP-1-1, PSP-1-2, and PSP-1-3 were determined by high-performance gel permeation chromatography (HPGPC) [17]. Accurately weighed 2 mg of each sample (PSP-1-1, PSP-1-2, and PSP-1-3) and dextran standards of varying molecular weights (5 × 10^3^, 1 × 10^4^, 2 × 10^4^, 5 × 10^4^, 1 × 10^5^, 2 × 10^5^, 4 × 10^5^, and 8 × 10^5^ Da) were dissolved in 1 mL of mobile phase to prepare 2 mg/mL solutions. After vortexing for 10 min, the solutions were centrifuged at 12,000 r/min for 10 min. The supernatant was collected and passed through a 0.22 µm filter membrane prior to injection. Analysis was performed on a high-performance liquid chromatography system equipped with a differential refractive index detector (Shimadzu, Japan) connected in series with TSKgel G5000 PWxl and TSKgel G3000 PWxl gel columns. A 20 µL aliquot was injected and eluted with pure water at a flow rate of 0.6 mL/min, with the column compartment maintained at 40 °C. A standard curve of lg (molecular weight) versus retention time (RT) was constructed to obtain the molecular weight calculation equation. The chromatograms and retention times were recorded, and the retention times were substituted into the equation to calculate the molecular weights of the polysaccharides.

### 2.4. FT-IR Analysis

FT-IR analysis followed the method of Wang et al. [17]. Accurately weigh 1.3 mg each of PSP-1-1, PSP-1-2, and PSP-1-3, together with 100 mg of potassium bromide, into a dried mortar and grind thoroughly. The mixture was then uniformly ground under an infrared lamp until no crystalline particles were observed, after which it was pressed into a thin pellet and placed in a desiccator. The sample was scanned using a Vertex 70 FT-IR spectrometer (Bruker, Billerica, MA, USA) at a resolution of 4 cm^−1^ over the range of 4000–400 cm^−1^.

### 2.5. Methylation Analysis

Based on the method reported by Ji et al. [18] with slight modifications, place 2 mg of the polysaccharide sample in a glass reaction vial. The sample was dissolved in 1.0 mL of anhydrous dimethyl sulfoxide, followed by the addition of 10 mg of dried sodium hydroxide powder. The mixture was reacted for 60 min in a 30 °C water bath under magnetic stirring. Then, 1.0 mL of iodomethane was added dropwise, and the mixture was further stirred at 30 °C for 1 h in the dark. The reaction was quenched by adding 2 mL of ultrapure water. The methylated polysaccharide was extracted with chloroform (3 × 2 mL); the combined organic phases were washed with ultrapure water, dried over anhydrous sodium sulfate, and concentrated under reduced pressure. The methylation procedure was repeated until complete methylation was confirmed. Complete methylation was verified by FT-IR spectroscopy, in which the O-H stretching absorption at approximately 3200–3600 cm^−1^ disappeared; if this absorption was still detectable, the methylation step was repeated. In addition, the final GC-MS profile showed no peaks corresponding to under-methylated alditol acetates. The methylated polysaccharide was then sequentially subjected to trifluoroacetic acid hydrolysis, sodium borodeuteride reduction, and acetic anhydride acetylation. The resulting acetylated product was analyzed by GC-MS using a Shimadzu GCMS-QP2010 Ultra system (Shimadzu Co., Ltd., Kyoto, Japan) equipped with an RXI-5 SIL MS column (30 m × 0.25 mm × 0.25 μm) and a quadrupole mass spectrometer with an electron ionization (EI) source. The injector temperature was set at 250 °C. The column temperature was initially held at 120 °C, then raised to 280 °C at a rate of 4 °C/min and maintained at 280 °C for 5 min. Helium served as the carrier gas at a flow rate of 1 mL/min. Mass spectrometry analysis was performed at the initial temperature of 140 °C for 2.0 min, and the temperature is increased to 230 °C by 3 °C/min for 3 min. The scan mode was SCAN with a range (*m/z*) from 50 to 350.

### 2.6. Cytotoxicity Assessment by MTT Assay

Cell viability was assessed using the MTT assay, which was adapted from the protocols of Sung et al. [19] and Xu et al. [20] with minor adjustments. NCI-H716 cells were maintained in a humidified 37 °C incubator under 5% CO_2_ in RPMI 1640 medium supplemented with 10% FBS, 100 IU/mL penicillin, and 100 µg/mL streptomycin. For the assay, 5 × 10^4^ cells per well were seeded in 96-well plates. The experimental design included three distinct well types: (i) cell-free blank wells, containing only culture medium without cells, to determine the background absorbance of the MTT reagent and medium; (ii) untreated cell control wells, containing cells and culture medium without polysaccharide treatment (Blank group), to establish the baseline viability (100%); and (iii) polysaccharide-treated wells, containing cells incubated with pumpkin seed polysaccharide at final concentrations of 12.5, 25, 50, 100, 200, and 400 µg/mL. All polysaccharide solutions were prepared in RPMI 1640 medium without FBS. PSP-1, PSP-1-1, PSP-1-2, PSP-1-3, and PSP-1s were tested separately in independent experiments. After gentle mixing, the plates were incubated at 37 °C under 5% CO_2_ for 24 h. At 16 h of culture, 20 µL of MTT reagent (5 mg/mL) was added to every well, including cell-free blanks and untreated controls, and the plates were incubated for the remaining 8 h. The MTT-containing solution was then carefully removed, and 200 µL of dimethyl sulfoxide (DMSO) was added to each well to dissolve the formazan crystals. Optical density was recorded at 570 nm using a Perkin-Elmer multi-function microplate reader (Waltham, MA, USA). Cell viability (%) was calculated as: (OD_treated−OD_cell-free blank)/(OD_untreated control−OD_cell-free blank) × 100.

### 2.7. Detection of GLP-1 Secretion in NCI-H716 Cells

The experiment was performed with slight modifications according to the method of Okimura et al. [21]. NCI-H716 cells in the logarithmic growth phase were collected and resuspended in DMEM complete medium. The cell suspension was seeded into 24-well plates at a density of 3 × 10^5^ cells per well and incubated in a humidified incubator at 37 °C with 5% CO_2_ for 48 h. PSP-1, PSP-1-1, PSP-1-2, PSP-1-3, and PSP-1s were individually dissolved in PBS to prepare working solutions at a concentration of 200 μg/mL, with the pH adjusted to 7.0. The old medium in the 24-well plates was discarded, and the prepared sample solutions were added. After 2 h of incubation, the supernatants were collected into 1.5 mL centrifuge tubes and immediately measured according to the biologically active GLP-1 kit instructions. A Blank group without polysaccharide treatment was set as the control.

### 2.8. Determination of Sweet Taste Receptor Signaling Pathway-Related Gene Expression in NCI-H716 Cells

Real-time quantitative polymerase chain reaction (RT-qPCR) was performed following the methods described by Bai et al. [22], Kim et al. [23], and Wang et al. [24]. Cell samples were harvested after 2 h of incubation and collection of the supernatant, as described in Section 2.7. Total RNA was extracted with an RC112 kit. The total RNA extracted from the cells was subsequently reverse-transcribed into complementary DNA (cDNA) following the protocol provided by the reverse transcription kit (R223). The obtained cDNA was amplified using a qPCR kit (Q711) and the designed primers, followed by real-time fluorescent quantitative detection (CFX Connect Real-Time Fluorescent Quantitative PCR Detection System, Bio-Rad Laboratories, Inc., Hercules, CA, USA). Using β-actin as the internal reference gene, the relative expression levels of the T1R2, T1R3, α-gustducin, phospholipase Cβ2 (PLCβ2), inositol 1,4,5-triphate receptor (IP3R), transient receptor potential melastatin 5 (TRPM5), glucose transporter 2 (GLUT2), and sodium-glucose cotransporter 1 (SGLT1) genes were calculated using the relative quantification method of 2^−ΔΔCt^. The primers designed are shown in Table S2.

### 2.9. Animal Experiment

All animal experimental procedures were approved by the Ethics Committee for Experimental Animals at South China Agricultural University (Animal Experiment Ethics Review No. 2024b151). All animal experiments were conducted in accordance with the ARRIVE guidelines (Animal Research: Reporting of In Vivo Experiments). A total of 36 male C57BL/6N mice, with body weights of 12–17 g, were reared in a specific pathogen-free facility at South China Agricultural University’s Animal Center (Certificate Number SYXK 2024-0136). Each animal was housed in an individual cage, and the housing conditions were maintained at a temperature range of 22 to 28 °C and humidity levels of 50 to 60%, with a light/dark cycle of 12 h each. The experimental methods were adapted and enhanced from Sun et al. [25] and Yang et al. [1]. The mice were randomly assigned using a computer-generated random sequence (1:5 allocation) to either a control group (Con group, *n* = 6), which received a standard diet (2530 kcal/kg), or a high-fat diet group (HFD group, *n* = 30), which received a high-fat diet (5240 kcal/kg). The dietary compositions are detailed in Table 1. Because the diets were visibly different, animal caretakers could not be blinded to diet allocation; however, they were not involved in outcome assessment. Outcome assessors and statisticians were blinded to group allocation.

After 4 weeks of dietary intervention, the HFD group received intraperitoneal injections of low-dose STZ at 50 mg/kg, while the Con group was administered citrate buffer (pH = 4.5) following an overnight fast. Two days after the intraperitoneal injection of STZ, fasting blood glucose (FBG) levels were measured. Mice with FBG levels > 14 mmol/L were considered successfully modeled. Mice that did not meet this criterion received a second intraperitoneal injection of STZ at 50 mg/kg. Two days later, FBG levels were measured again, and mice with FBG > 14 mmol/L were considered successfully modeled. After all mice in the high-sugar/high-fat diet group were successfully modeled, they were further randomly assigned to five subgroups (*n* = 6 per group): the model group (Model group), the metformin group (Met group, 100 mg/kg/day), the low-dose PSP-1s group (L-PSP-1s group, 50 mg/kg/day), the medium-dose PSP-1s group (M-PSP-1s group, 100 mg/kg/day), and the high-dose PSP-1s group (H-PSP-1s group, 200 mg/kg/day). After establishing the model, mice in the Con and Model groups received normal saline via oral gavage for 4 weeks, while the mice in the other experimental groups were administered the respective substances through gavage. Throughout the study, the body weight, food consumption, and water intake of the mice in each group were monitored bi-daily. Additionally, FBG levels were assessed weekly, and oral glucose tolerance tests (OGTT) were administered to the mice one day before the end of the experiment. At the end of the experiment, after an overnight fast, the mice were deeply anesthetized with 3% isoflurane and euthanized by cervical dislocation. Death was confirmed by the absence of pedal withdrawal and corneal reflexes and by the cessation of respiration and heartbeat. Blood samples and tissue samples from the pancreas and hypothalamus, as well as colonic contents, were then collected for analysis.

### 2.10. Determination of FBG Level

Following a 12 h fasting period, tail-vein blood was collected from mice across all experimental groups. The FBG levels were subsequently measured utilizing a glucometer (ACCU-CHEK Guide Me, Roche; Shanghai, China) in conjunction with glucose testing strips, in accordance with the manufacturer’s protocol.

### 2.11. OGTT

The methods developed by Sun et al. [25] were referenced. Following a 12 h fasting period, the mice in each group received an intragastric administration of glucose at a dosage of 2 g/kg body weight. Subsequently, blood glucose levels were measured at 0, 30, 60, and 120 min post-glucose administration, utilizing the methodology outlined in Section 2.10. The corresponding area under the curve (AUC) was then calculated as follows:

AUC[mmol/(L·h)] = (BG_0_ + BG_30_)/2 × 0.5 + (BG_30_ + BG_60_)/2 × 0.5 + (BG_60_ + BG_120_)/2 × 1 (1)

where BG_0_, BG_30_, BG_60_, and BG_120_ denote the blood glucose levels measured at 0, 30, 60, and 120 min, respectively.

### 2.12. Pancreatic Pathology Analysis

Referring to the method of Wang et al. [26], the mice pancreas was fixed in 10% neutral formalin for 24 h, then washed with phosphate-buffered saline, dehydrated with gradient ethanol, cleared with xylene, embedded in paraffin, sectioned, and mounted on slides. After drying, the samples were dewaxed, rehydrated, stained with HE, dehydrated again, cleared, sealed with neutral balsam, and examined under a microscope.

### 2.13. Serum Biochemical Analysis

The blood samples collected were subjected to centrifugation at 3000 r/min for 10 min at 4 °C. The resultant serum was subsequently stored at −80 °C until further analysis. Serum concentrations of TC, TG, LDL-C, HDL-C, GSP, insulin, and GLP-1 were quantified using commercial kits in accordance with the manufacturer’s protocols. The homeostasis model assessment of insulin resistance (HOMA-IR) and the quantitative insulin sensitivity check index (QUICKI) were calculated as follows:

HOMA-IR = [insulin (μIU/mL) × FBG (mmol/L)]/22.5(2)

QUICKI = 1/{log [insulin (μIU/mL)] + log [FBG (mg/dL)]}(3)

### 2.14. Determination of SCFAs Level in Colonic Contents

The concentrations of six SCFAs, namely acetic acid, propionic acid, butyric acid, isobutyric acid, valeric acid, and isovaleric acid, in mouse colonic contents were determined by GC-MS with reference to Zhu et al. [27]. Briefly, 50 mg of colonic contents was mixed with 10 μL of 50% phosphoric acid and 600 μL of anhydrous diethyl ether. The mixture was shaken for 30 min and centrifuged at 12,000 r/min for 10 min at 4 °C. The diethyl ether layer was transferred to a tube containing 10 mg of anhydrous sodium sulfate to remove residual water and filtered through a 0.22 μm organic solvent-compatible membrane before analysis.

Analysis was performed using an Agilent HP6890-5973 GC-MS system equipped with a capillary column (60 m × 0.25 mm × 0.25 μm) (Agilent 122-7062, Agilent Technologies, Santa Clara, CA, USA). The oven temperature was initially set at 90 °C, increased to 160 °C at 15 °C/min and held for 5 min, and then increased to 210 °C at 15 °C/min and held for 5 min. Nitrogen was used as the carrier gas at a flow rate of 1.0 mL/min. The inlet temperature was 250 °C, and a 5 μL aliquot was injected in splitless mode. The detector temperature was set at 210 °C. Mass spectra were acquired over an *m/z* range of 20–300.

SCFAs were quantified using external calibration. A mixed SCFA standard stock solution at 10 mg/mL was prepared in diethyl ether and diluted to 1 mg/mL. Working standard solutions at 6.25, 12.5, 25, 50, 100, and 200 μg/mL were subsequently prepared by serial dilution in diethyl ether. Each SCFA was quantified using its corresponding calibration curve constructed by plotting peak area against standard concentration (Table S3).

Recovery experiments were performed by spiking blank colonic content samples with SCFA standards at low, medium, and high concentration levels (*n* = 3 per level). The extraction recovery was calculated as the ratio of the peak area of SCFA in the pre-extraction spiked sample to that in the post-extraction spiked sample, multiplied by 100%. Recovery values in the range of 70–120% were considered acceptable. Recovery rates for the six SCFAs ranged from 80.8% to 108.8%, with intraday and interday precision (RSD) below 5.0%.

### 2.15. 16S rRNA Gut Microbiota Analysis

The gut microbiota analysis was conducted using the approach described by Chen et al. [12]. Microbial DNA was isolated from mouse colonic contents using a DNeasy PowerSoil kit (Qiagen, Hilden, Germany) following the manufacturer’s instructions. After DNA quality assessment, the V3-V4 hypervariable regions of the bacterial 16S rRNA gene were amplified with universal primers 343F (5′-TACGGRAGGCAGCAG-3′) and 798R (5′-AGGGTATCTAATCCT-3′), and the purified amplicons were submitted to OE Biotech Co., Ltd. (Shanghai, China) for sequencing on an Illumina NovaSeq 6000 platform with paired-end 250 bp reads. Raw FASTQ reads were preprocessed with cutadapt to detect and remove adapters; after trimming, low-quality reads were filtered, denoised, merged, and chimeras were removed using DADA2 with the default parameters in QIIME 2 (2020.11). Representative sequences for each Amplicon Sequence Variant (ASV) were selected using QIIME 2 and annotated against the Silva database (version 138) using the q2-feature-classifier plugin with default settings.

### 2.16. Determination of Hypothalamic GLP-1R Protein Expression in Mice

Western blotting (WB) was performed with reference to Chen et al. [28] to determine GLP-1R protein expression in the hypothalamus. Briefly, 25 mg of hypothalamic tissue was homogenized in 300 μL of ice-cold RIPA lysis buffer supplemented with protease inhibitors. The homogenate was incubated on ice for 10 min and centrifuged at 12,000 r/min for 15 min at 4 °C. The supernatant was collected, and protein concentrations were determined using a commercial protein assay kit. A 200 μL aliquot of the protein supernatant was mixed with 5× loading buffer at a 4:1 (*v*/*v*) ratio and heated in boiling water for 5 min. Proteins were separated by 10% (*w*/*v*) SDS-polyacrylamide gel electrophoresis. The gel was cut according to the molecular weights of the target proteins, and proteins were transferred onto PVDF membranes at a constant current of 300 mA for 75 min for GLP-1R and 60 min for β-actin.

The membranes were blocked with 5% non-fat milk in PBST for 90 min at room temperature and incubated overnight at 4 °C with rabbit anti-GLP-1R or mouse anti-β-actin primary antibodies. After washing three times with PBST for 15 min each, the membranes were incubated with the corresponding HRP-conjugated goat anti-rabbit IgG secondary antibodies for 90 min at room temperature. The membranes were then washed three times with PBST for 10 min each. Protein bands were visualized using an ECL substrate and recorded with a gel imaging system. β-actin served as the loading control.

### 2.17. Determination of Appetite-Related Gene Expression in Mice

RT-qPCR was conducted with minor modifications based on the methodologies outlined by Bai et al. [22] and Lin et al. [29]. Briefly, hypothalamus tissue was collected and transferred to a mortar pre-cooled with liquid nitrogen, then ground into a fine powder. An appropriate amount of the powder was then transferred to a 1.5 mL centrifuge tube containing lysis buffer, and immediately vortexed at high speed or pipetted repeatedly until no obvious precipitate remained in the lysis buffer. The lysate was placed on ice for 2 min, then centrifuged at 12,000 r/min at 4 °C for 5 min. The supernatant was carefully aspirated into a new 1.5 mL centrifuge tube, and the subsequent procedure was carried out according to the method described in Section 2.8. Using β-actin as the internal reference gene, the relative expression levels of the Proopiomelanocortin (POMC), Cocaine and Amphetamine-Regulated Transcript (CART), Neuropeptide Y (NPY), and Agouti-Related Protein (AgRP) genes were calculated using the relative quantification method of 2^−ΔΔCt^. The primers designed are shown in Table S4.

### 2.18. Statistical Analysis

All experiments were performed with at least three technical replicates. Cell experiments included four biological replicates per group, and animal experiments included six biological replicates per group. All data are presented as mean ± standard deviation (SD). Repeated-measures Analysis of Variance (ANOVA) was implemented for body weight, food intake, water intake, and FBG, which were measured multiple times in the same animals, while one-way ANOVA was implemented for other data using SPSS 25.0 software (SPSS Inc., Chicago, IL, USA). *p* < 0.05 represented a statistical difference, while *p* < 0.01 indicated a more significant statistical difference.

## 3. Results and Discussion

### 3.1. Molecular Weight of Pumpkin Seed Polysaccharides

**Figure 1:**
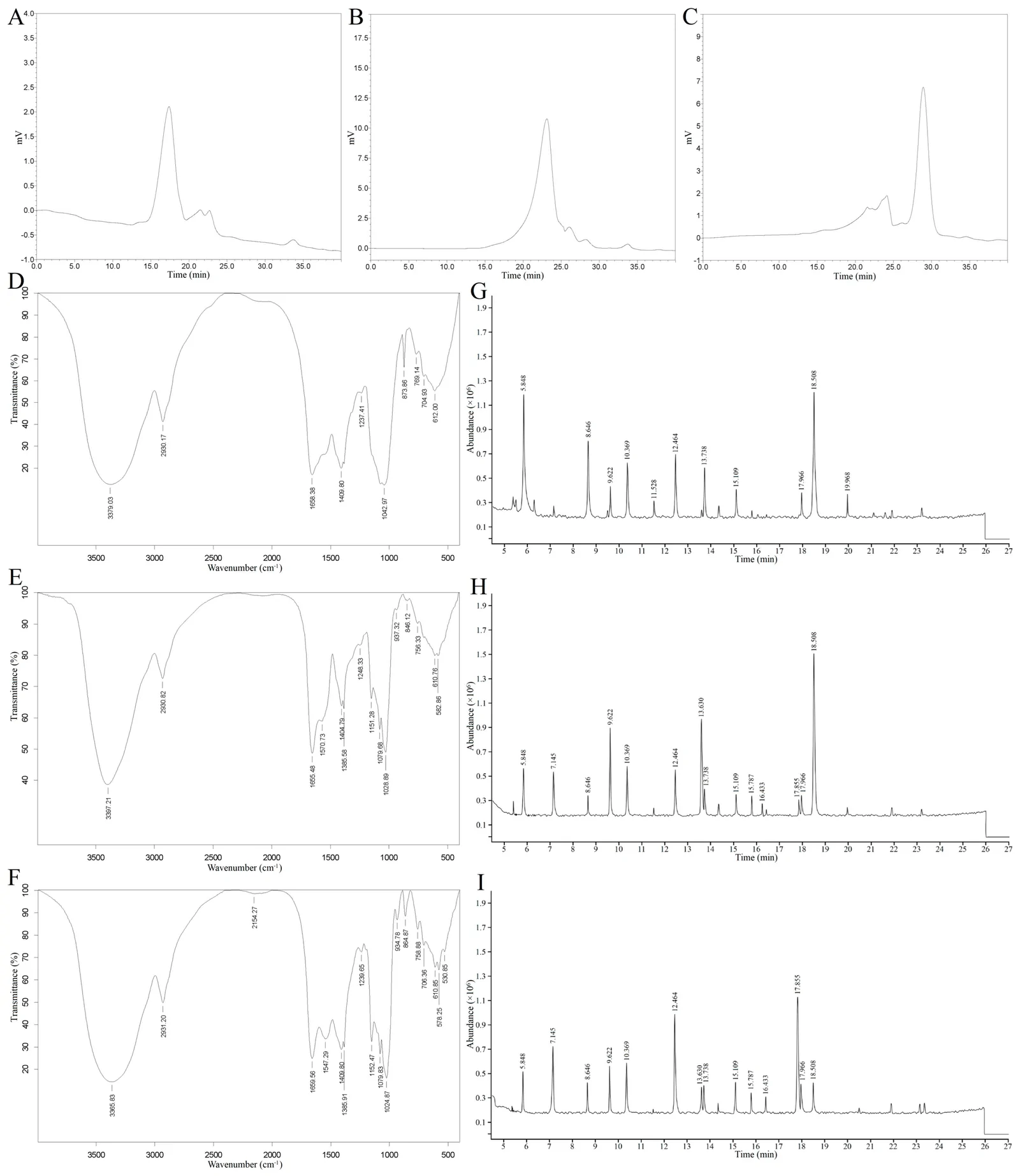
Molecular weight determination of pumpkin seed polysaccharides: PSP-1-1 (**A**), PSP-1-2 (**B**), and PSP-1-3 (**C**); FT-IR spectra of pumpkin seed polysaccharides: PSP-1-1 (**D**), PSP-1-2 (**E**), and PSP-1-3 (**F**); Methylation analysis total ion chromatograms of pumpkin seed polysaccharides: PSP-1-1 (**G**), PSP-1-2 (**H**), and PSP-1-3 (**I**).

As shown in Figure 1A–C, PSP-1-1, PSP-1-2, and PSP-1-3 each exhibited a single main symmetrical peak, with the main peak accounting for 97%, 93%, and 89% of the total peak area, respectively, suggesting relatively narrow molecular-weight distributions and high apparent chromatographic purity by HPGPC. The main-peak retention times were 17.39, 23.11, and 28.91 min, respectively. Using the standard curve derived from dextran standards, the weight-average molecular weights were calculated to be 29,797 Da, 11,209 Da, and 4346 Da, respectively.

### 3.2. FT-IR Analysis of Pumpkin Seed Polysaccharides

As shown in Figure 1D–F, all three pumpkin seed polysaccharide fractions exhibited a broad and intense absorption peak around 3360–3400 cm^−1^, which is a characteristic absorption peak of polysaccharides, arising from the stretching vibration of O-H groups in the polysaccharide chains. This also indicates the presence of hydrogen bonds caused by O-H groups within or between polysaccharide molecules, resulting in peak broadening [30]. Meanwhile, a characteristic absorption peak was observed around 2930 cm^−1^, attributed to the stretching vibration of alkane C-H bonds, further confirming the polysaccharide nature [31]. An absorption peak appeared around 1658 cm^−1^, caused by the asymmetric and symmetric stretching vibrations of C=O, indicating the presence of C=O functional groups. Additionally, the peak around 1410 cm^−1^ is associated with C-O stretching vibrations, suggesting the existence of uronic acids [17]. Weak absorption peaks around 1570 cm^−1^ for PSP-1-2 and PSP-1-3 indicate the presence of N-H functional groups, suggesting a small amount of protein bound to the polysaccharides [17]. Absorption peaks in the range of 1200–1000 cm^−1^ are attributable to pyranose rings, resulting from the stretching vibration of C-O-H in the ether linkage C-O-C, indicating that all three fractions contain pyranose sugars [32]. The peak near 920 cm^−1^ corresponds to D-pyranoglucosyl groups, which is consistent with the monosaccharide composition results showing that PSP-1-2 and PSP-1-3 have higher glucose content [33]. Furthermore, PSP-1-1 showed absorption peaks at 874 cm^−1^ and 769 cm^−1^, assigned to α- and β-glycosidic linkages, respectively. In addition, PSP-1-1 displayed two peaks in the range of 1100–1010 cm^−1^, suggesting the possible presence of furanose residues. In other words, PSP-1-1 may simultaneously contain α- and β-glycosidic linkages as well as both pyranose and furanose ring forms. For PSP-1-2 and PSP-1-3, peaks near 860 cm^−1^ and 937 cm^−1^ were also observed, indicating the possible presence of α- and β-glycosidic linkages as well [34].

### 3.3. Methylation Analysis of Pumpkin Seed Polysaccharides

According to our group’s previous study [5], pumpkin seed polysaccharides were mainly composed of galactose (Gal), arabinose (Ara), glucose (Glc), and xylose (Xyl). The relative molar ratios of these four monosaccharides were 61.5:24.4:5.9:0.5 for PSP-1-1, 38.2:14.8:28.6:8.9 for PSP-1-2, and 38.8:14.2:31.7:7.3 for PSP-1-3. Methylation analysis was further performed to verify their monosaccharide compositions and elucidate the configurations of their glycosidic linkages.

As shown in Figure 1G and Table S5, PSP-1-1 was composed of 11 types of glycosidic linkages: Ara(f)-(1→, Gal(p)-(1→, Glc(p)-(1→, →5)-Ara(f)-(1→, →2,3)-Rha(p)-(1→, →3)-Gal(p)-(1→, →4)-Gal(p)-(1→, →6)-Gal(p)-(1→, →4,6)-Gal(p)-(1→, →3,6)-Gal(p)-(1→, and →1,3,6)-Gal(p)-(1→, with molar percentages of 20.82%, 13.04%, 5.56%, 9.93%, 1.56%, 10.70%, 8.75%, 3.75%, 2.61%, 20.85%, and 2.43%, respectively. The main constituent monosaccharide residues were arabinose and galactose, whose combined proportion was close to the monosaccharide composition, thereby verifying the monosaccharide composition results. Among the arabinose residues, the predominant form was terminal residues, indicating that most branches of this polysaccharide were terminated by arabinose. Additionally, approximately 10% of →5)-Ara(f)-(1→ was present, which was speculated to constitute part of the branches. For the other major constituent monosaccharide, galactose, the main residues were Gal(p)-(1→, →3)-Gal(p)-(1→, →4)-Gal(p)-(1→, and →3,6)-Gal(p)-(1→, indicating that galactose exhibited diverse glycosidic linkage patterns and may constitute the main chain.

As shown in Figure 1H and Table S6, PSP-1-2 was composed of 14 types of glycosidic linkages: Ara(f)-(1→, Xyl(p)-(1→, Gal(p)-(1→, Glc(p)-(1→, →5)-Ara(f)-(1→, →3)-Gal(p)-(1→, →4)-Glc(p)-(1→, →4)-Gal(p)-(1→, →6)-Gal(p)-(1→, →3,4)-Glc(p)-(1→, →2,4)-Glc(p)-(1→, →4,6)-Glc(p)-(1→, →4,6)-Gal(p)-(1→, and →3,6)-Gal(p)-(1→, with molar percentages of 8.22%, 7.69%, 1.97%, 14.63%, 8.65%, 8.07%, 15.20%, 3.18%, 2.09%, 1.92%, 1.77%, 1.36%, 1.94%, and 23.31%, respectively. The main monosaccharide residues constituting the polysaccharide were arabinose, xylose, galactose, and glucose, and their combined proportion was close to the monosaccharide composition, thereby verifying the monosaccharide composition results. In PSP-1-2, arabinose mainly existed as Ara(f)-(1→ and →5)-Ara(f)-(1→; xylose mainly existed as Xyl(p)-(1→; galactose mainly existed as →3)-Gal(p)-(1→ and →3,6)-Gal(p)-(1→; and glucose mainly existed as Glc(p)-(1→ and →4)-Glc(p)-(1→. Accordingly, it was inferred that the main terminal residues of PSP-1-2 were Ara(f)-(1→, Xyl(p)-(1→, and Glc(p)-(1→, and the main chain was likely composed of →5)-Ara(f)-(1→, →3)-Gal(p)-(1→, →4)-Glc(p)-(1→, and →3,6)-Gal(p)-(1→.

As shown in Figure 1I and Table S7, PSP-1-3 was composed of 14 types of glycosidic linkages: Ara(f)-(1→, Xyl(p)-(1→, Gal(p)-(1→, Glc(p)-(1→, →5)-Ara(f)-(1→, →3)-Gal(p)-(1→, →4)-Glc(p)-(1→, →4)-Gal(p)-(1→, →6)-Gal(p)-(1→, →3,4)-Glc(p)-(1→, →2,4)-Glc(p)-(1→, →4,6)-Glc(p)-(1→, →4,6)-Gal(p)-(1→, and →3,6)-Gal(p)-(1→, with molar percentages of 7.38%, 11.57%, 4.70%, 8.21%, 8.80%, 16.78%, 2.86%, 3.69%, 4.94%, 1.97%, 1.52%, 19.33%, 3.79%, and 4.46%, respectively. The main monosaccharide residues constituting the polysaccharide were arabinose, xylose, galactose, and glucose, and their combined proportion was close to the monosaccharide composition, thereby verifying the monosaccharide composition results. In PSP-1-3, the glycosidic linkage types of arabinose were similar to those of PSP-1-2, mainly existing as Ara(f)-(1→ and →5)-Ara(f)-(1→; xylose was also similar to PSP-1-2, mainly existing as Xyl(p)-(1→; galactose mainly existed as →3)-Gal(p)-(1→, and the →3,6)-Gal(p)-(1→ linkage that had a relatively high molar percentage in PSP-1-2 was present at a very low proportion in PSP-1-3, whereas glucose mainly existed as Glc(p)-(1→ and →4,6)-Glc(p)-(1→, which differed from PSP-1-2. Thus, it could be inferred that the main terminal residues of PSP-1-3 were Ara(f)-(1→, Xyl(p)-(1→, and Glc(p)-(1→, and the main chain was likely composed of →5)-Ara(f)-(1→, →3)-Gal(p)-(1→, and →4,6)-Glc(p)-(1→.

It was found that the three pumpkin seed polysaccharides shared certain structural similarities: their main residue compositions all contained →5)-Ara(f)-(1→ and →3)-Gal(p)-(1→, and all possessed terminal residues composed of Ara(f)-(1→ and Glc(p)-(1→. For instance, the arabinogalactan with hypoglycemic activity isolated from the tuberous cortex of white-skinned sweet potato by Ozaki et al. [35] also contained →5)-Ara(f)-(1→ and Ara(f)-(1→ structures. PSP-1-1 additionally contained Gal(p)-(1→, whereas PSP-1-2 and PSP-1-3 had low levels of Gal(p)-(1→ and exhibited Xyl(p)-(1→ terminal residues. The main chains of PSP-1-1 and PSP-1-2 may contain →3,6)-Gal(p)-(1→ structures; similarly, He et al. [36] found that the main chain of the hypoglycemic polysaccharide from the peel of wampee was composed of →3,6)-Gal(p)-(1→. The main chain of PSP-1-3 may be composed of →4,6)-Glc(p)-(1→, a structure also found in the hypoglycemic polysaccharide from *Polygonum cuspidatum* [37]. Furthermore, the →4)-Glc(p)-(1→ structure present in PSP-1-2 is also an important backbone in the hypoglycemic polysaccharides from Lanzhou lily [38] and *Polygonum cuspidatum* [37].

### 3.4. Cytotoxic Effects of Pumpkin Seed Polysaccharides on NCI-H716 Cells

**Figure 2:**
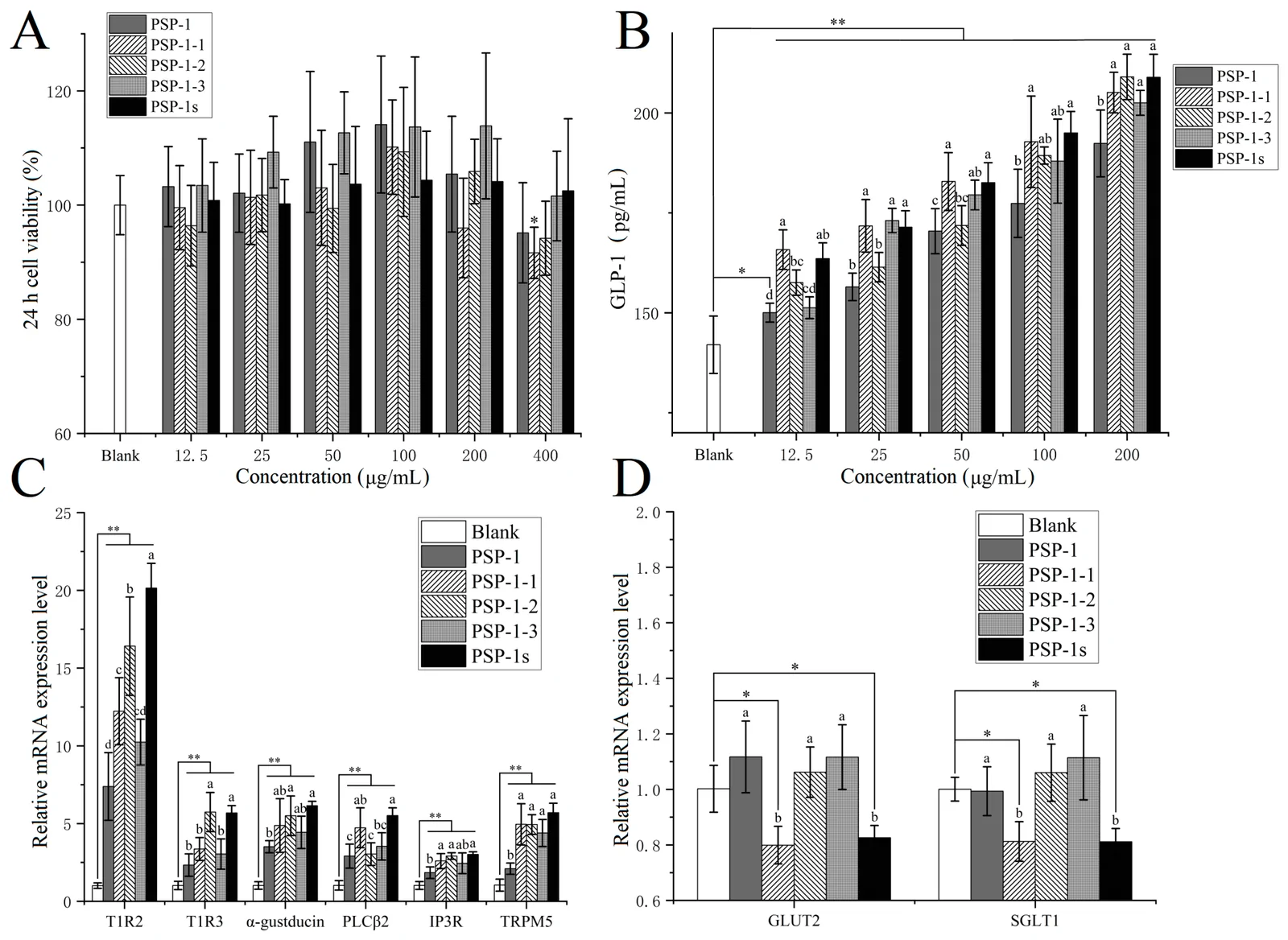
Effects of pumpkin seed polysaccharide fractions on NCI-H716 cell viability (**A**), GLP-1 secretion (**B**), and the expression of sweet taste receptor signaling pathway-related genes: T1R2, T1R3, α-gustducin, PLCβ2, IP3R, TRPM5 (**C**); GLUT2, SGLT1 (**D**). Different letters indicate significant differences between groups (*p* < 0.05); * indicates significant difference compared with the Blank group (0.01 ≤ *p* < 0.05); ** indicates extremely significant difference compared with the Blank group (*p* < 0.01).

As illustrated in Figure 2A, within the concentration range of 12.5–200 μg/mL, none of the pumpkin seed polysaccharide fractions exhibited cytotoxicity after 24 h of treatment; cell viability was not significantly lower than that of the Blank group (*p* > 0.05). However, when the concentration was increased to 400 μg/mL, the cell viability of the PSP-1-1 group dropped to 90.56%, which was significantly lower than that of the Blank group (*p* < 0.05). Based on these cytotoxicity assessment results, the upper concentration limit for subsequent experiments was set at 200 μg/mL to ensure that the experimental system remained within the non-toxic range.

### 3.5. Effects of Pumpkin Seed Polysaccharides on GLP-1 Secretion in NCI-H716 Cells

As shown in Figure 2B, PSP-1, PSP-1-1, PSP-1-2, PSP-1-3, and PSP-1s all began to exhibit GLP-1 secretion-promoting activity at a concentration of 12.5 μg/mL. Within the concentration range of 12.5–200 μg/mL, the stimulatory effects of all pumpkin seed polysaccharide fractions on GLP-1 secretion from NCI-H716 cells were dose-dependent. Specifically, at a concentration of 200 μg/mL, compared with the Blank group, the GLP-1 secretion levels of the above polysaccharide groups were significantly increased by 35.45%, 44.41%, 47.17%, 42.61%, and 47.10%, respectively (*p* < 0.05). Meanwhile, there were no significant differences in GLP-1 secretion among the PSP-1-1, PSP-1-2, PSP-1-3, and PSP-1s groups (*p* > 0.05); however, all of these groups were significantly higher than the PSP-1 group (*p* < 0.05), indicating that the biological activity of pumpkin seed polysaccharide fractions was enhanced after purification. A similar finding was reported by Wang et al. [39], in which two purified polysaccharide fractions from *Inonotus obliquus* polysaccharides exhibited significantly higher α-glucosidase inhibitory activity than the unpurified fraction.

### 3.6. Effects of Pumpkin Seed Polysaccharides on Gene Expression in NCI-H716 Cells

The signaling of sweet taste receptors facilitates glucose absorption and stimulates the synthesis and secretion of GLP-1 in intestinal endocrine L cells [40]. As shown in Figure 2C, all pumpkin seed polysaccharide fractions increased the gene expression levels of the sweet taste receptors T1R2 and T1R3 in NCI-H716 cells. Compared with the Blank group, the T1R2 expression levels in the PSP-1, PSP-1-1, PSP-1-2, PSP-1-3, and PSP-1s groups were increased by 6.32-, 11.11-, 15.25-, 9.14-, and 18.95-fold, respectively (*p* < 0.01), while the T1R3 expression levels were increased by 1.28-, 2.30-, 4.63-, 1.98-, and 4.57-fold, respectively (*p* < 0.01). These results confirm that all pumpkin seed polysaccharide fractions promoted the gene expression of sweet taste receptors on L cells, and further demonstrate that the biological activity of the polysaccharides was enhanced after purification. A similar finding was reported by Yang et al. [16], who showed that the gene expression levels of intestinal sweet taste receptors T1R2 and T1R3 were decreased in diabetic rats, whereas *Astragalus* polysaccharides could increase the expression of T1R2 and T1R3, which was identified as a key mechanism underlying the hypoglycemic effect of this polysaccharide.

Figure 2C also showed that, compared with the Blank group, the α-gustducin expression levels in the PSP-1, PSP-1-1, PSP-1-2, PSP-1-3, and PSP-1s groups were increased by 2.45-, 3.79-, 4.40-, 3.36-, and 5.04-fold, respectively (*p* < 0.01); the PLCβ2 expression levels were increased by 1.83-, 3.60-, 1.96-, 2.44-, and 4.37-fold, respectively (*p* < 0.01); the IP3R expression levels were increased by 0.61-, 1.55-, 1.85-, 1.40-, and 1.96-fold, respectively (*p* < 0.01); and the TRPM5 expression levels were increased by 1.02-, 3.75-, 3.74-, 3.21-, and 4.47-fold, respectively (*p* < 0.01). These results indicate that all pumpkin seed polysaccharide fractions significantly upregulated the gene expression levels of α-gustducin, PLCβ2, IP3R, and TRPM5 in NCI-H716 cells (*p* < 0.01), suggesting that the α-gustducin/PLCβ2/IP3R/TRPM5 signaling cascade may be involved in their effects. α-Gustducin is a G protein that serves as a downstream signaling molecule of the sweet taste receptors T1R2 and T1R3, and is involved in sweet, umami, and bitter taste receptor signal transduction [4,16]. Jang et al. [41] observed in α-gustducin gene knockout mice that GLP-1 secretion was significantly reduced after oral glucose administration, and the regulation of plasma insulin and glucose levels was impaired. Further studies on isolated mouse intestines confirmed that the absence of α-gustducin severely impaired the intestinal GLP-1 secretory response to glucose stimulation. Upon activation, α-gustducin further activates PLCβ2 [42], which generates IP3. IP3 binds to the Ca^2+^ channel IP3R on the endoplasmic reticulum, leading to the release of Ca^2+^ from the endoplasmic reticulum into the cytoplasm [43,44]. The elevated intracellular Ca^2+^ concentration directly activates the Ca^2+^-sensitive monovalent cation channel TRPM5 (primarily permeable to Na^+^ and K^+^), causing Na^+^ influx and generating a depolarizing receptor potential [45,46]. When depolarization reaches a certain threshold, voltage-gated sodium channels (VGSCs) open, generating an action potential. The action potential propagates along the cell membrane, causing voltage-gated calcium channels (VGCCs) to open, through which extracellular Ca^2+^ rapidly flows into the cell, bringing the cytoplasmic Ca^2+^ concentration to its peak. The sharply elevated cytoplasmic Ca^2+^ then acts as the final messenger, triggering GLP-1-containing secretory vesicles to translocate toward the cell membrane. The concurrent increases in GLP-1 secretion and the mRNA expression of sweet taste receptor-related signaling components suggest that this pathway may be involved in the cellular response to pumpkin seed polysaccharides. However, these findings do not establish receptor activation or pathway dependence.

GLUT2 and SGLT1 are important glucose transporters in the intestine. SGLT1 forms a ternary complex with Na^+^ and glucose, utilizing the Na^+^ electrochemical gradient to transport glucose into the cell against its concentration gradient via secondary active transport, whereas GLUT2 facilitates glucose entry into the cell through passive transport. Together, they maintain glucose metabolic homeostasis [47]. Diabetes has been shown to cause abnormally increased expression of GLUT2 and SGLT1 in the intestine [48]. Lin et al. [49] found that in vitro fecal bacterial fermentation of flaxseed polysaccharides downregulated the mRNA and protein expression levels of GLUT2 and SGLT1 in Caco-2 cells, thereby effectively inhibiting glucose uptake in this cell model. In the present study, Figure 2D showed that, compared with the Blank group, the GLUT2 gene expression levels in the PSP-1-1 and PSP-1s groups were significantly decreased by 20.26% and 17.56%, respectively (*p* < 0.05), while the SGLT1 gene expression levels were significantly decreased by 18.80% and 18.92%, respectively (*p* < 0.05). These results suggest that these two pumpkin seed polysaccharide fractions may also influence cellular metabolic function by suppressing the expression of glucose transporter-related genes. In contrast, PSP-1, PSP-1-2, and PSP-1-3 had no significant effects on the gene expression levels of GLUT2 and SGLT1 in NCI-H716 cells (*p* > 0.05). A similar phenomenon was reported by Yang et al. [50], who found that *Polygonatum odoratum* polysaccharides, which promoted GLP-1 secretion, had no significant effect on the mRNA expression of intestinal GLUT2 and SGLT1 in diabetic rats.

Taken together, pumpkin seed polysaccharide treatment increased GLP-1 secretion and upregulated the mRNA expression of sweet taste receptors and associated signaling components in NCI-H716 cells. These findings suggest a possible involvement of sweet taste receptor-related signaling, although functional studies are required to establish its contribution to GLP-1 secretion. PSP-1-1 and PSP-1s also reduced GLUT2 and SGLT1 mRNA expression. The functional consequences of these transcriptional changes remain to be determined.

Furthermore, the combined collected fraction PSP-1s induced significantly higher T1R2 gene expression than all other treatment groups (*p* < 0.05) and exhibited the highest mean gene expression levels of α-gustducin, PLCβ2, IP3R, and TRPM5 among all groups, with no significant difference from the purified fraction PSP-1-1 (*p* > 0.05). Additionally, the GLUT2 and SGLT1 gene expression levels in the PSP-1s group were comparable to those in the PSP-1-1 group (*p* > 0.05) and significantly lower than those in all other treatment groups (*p* < 0.05). Together with its GLP-1 secretion-promoting activity, these gene expression results supported the selection of the combined collected fraction PSP-1s for subsequent animal experiments.

### 3.7. Effects of PSP-1s on Body Weight, Food Consumption, and Water Intake in Diabetic Mice

**Figure 3:**
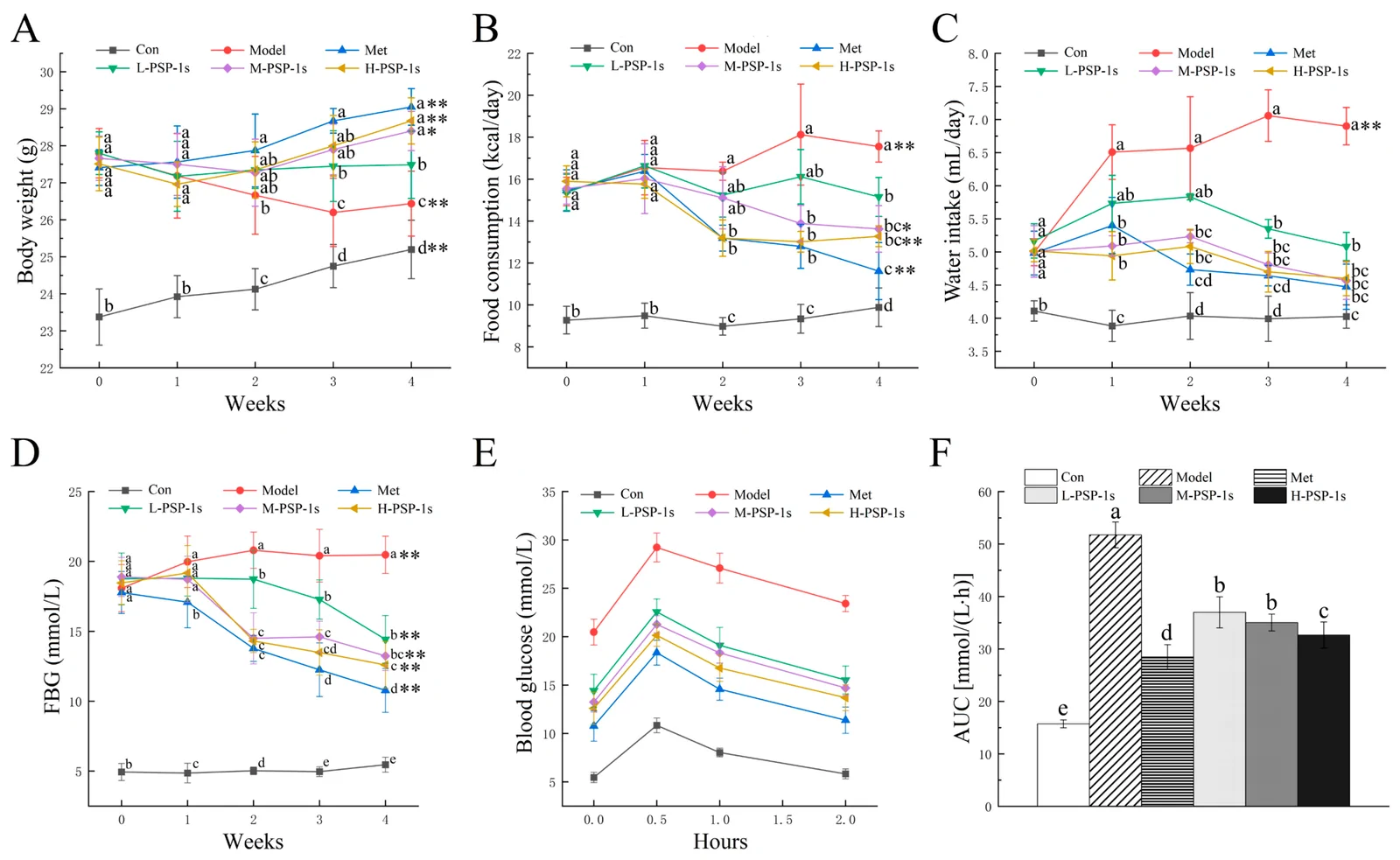
Effect of PSP-1s on (**A**) body weight, (**B**) food consumption, (**C**) water intake, (**D**) FBG level, (**E**) blood glucose curve during the OGTT, and (**F**) AUC of diabetic mice. Different letters represent significant differences in all groups (*p* < 0.05). * represents significant differences between the data at week 4 and week 0: * 0.01 ≤ *p* < 0.05, ** *p* < 0.01. *n* = 6.

As depicted in Figure 3A, over the course of the 4-week period, the body weight trajectory of the Con group demonstrated an overall upward trend. Conversely, the Model group mice experienced a weekly reduction in body weight, which was significantly lower than the pre-intervention level (week 0) by week 4 (*p* < 0.01), and also significantly lower than that of mice in all experimental groups except the Con group (*p* < 0.05). Figure 3B,C demonstrates that the diabetic mice exhibited a marked increase in both food consumption and water intake compared to the mice in the Con group. (*p* < 0.05), indicating that STZ-induced modeling had already impacted the physiological activities of the mice. At week 4, the food consumption and water intake of mice in the Model group were significantly higher than those in all other groups (*p* < 0.05). This pattern aligns with findings from pertinent studies. In diabetic mice, accelerated lipid degradation and enhanced protein catabolism contribute to muscle atrophy and weight loss [51]. Regarding food and water intake, diminished glucose uptake by peripheral tissues, such as muscle and adipose tissue, results in an inadequate cellular energy supply, thereby stimulating appetite through hypothalamic feeding centers, such as the activation of NPY/AgRP neurons [52]. Additionally, osmotic diuresis caused by hyperglycemia exacerbates polydipsia [53].

Following the administration of PSP-1s at doses of 100 or 200 mg/kg, the body weight of the diabetic mice initially decreased during the first week but subsequently recovered, ultimately surpassing the pre-intervention levels by week 4 (*p* < 0.05 and <0.01, respectively), while their food consumption decreased markedly (*p* < 0.05 and <0.01, respectively). Moreover, the water intake of mice across all groups receiving varying doses of PSP-1s demonstrated an initial increase, followed by a decrease over the 4-week period. These findings suggest that PSP-1s can mitigate weight loss and ameliorate symptoms of polyphagia and polydipsia in diabetic mice.

### 3.8. Effects of PSP-1s on FBG and Glucose Tolerance in Diabetic Mice

As depicted in Figure 3D, the FBG levels of mice in the Con group remained within the normal physiological range, while those on a high-glucose/high-fat diet with STZ had FBG levels above 14 mmol/L, meeting the criteria for experimental research. Beginning in week 2, the FBG levels in mice from the Model group were significantly elevated compared to all other groups (*p* < 0.05) and showed a marked increase from pre-intervention levels by week 4 (*p* < 0.01). By week 4, the FBG levels of mice in the Met group, as well as those receiving various doses of PSP-1s, exhibited a significant decrease compared to pre-intervention levels (all *p* < 0.01). Metformin demonstrated the most pronounced effect, followed sequentially by PSP-1s at doses of 200 mg/kg, 100 mg/kg, and 50 mg/kg. Among them, intervention with 200 mg/kg PSP-1s resulted in a reduction of FBG in diabetic mice from 18.46 ± 1.58 mmol/L pre-intervention to 12.60 ± 1.67 mmol/L. These findings suggest that PSP-1s effectively reduces FBG in type 2 diabetic mice, with the efficacy of FBG improvement becoming more pronounced with increasing doses of PSP-1s. A similar phenomenon was reported by Wang et al. [54]. They discovered that a polysaccharide from the *Gynostemma pentaphyllum* herb reduced FBG levels in a dose-dependent manner over 4 weeks.

Glucose tolerance pertains to the body’s capacity to regulate blood glucose levels following the ingestion of external glucose sources. A greater degree of impaired glucose tolerance is associated with an increased AUC [55]. As illustrated in Figure 3E,F, the AUC value for mice in the Model group was significantly elevated compared to each of the experimental groups (*p* < 0.05). The AUC values were reduced in both the Met group and the groups administered varying doses of PSP-1s (*p* < 0.05). Within the PSP-1s groups, the mice in the H-PSP-1s group exhibited the most pronounced improvement in glucose tolerance, with their AUC value being 36.93% lower than that of the Model group. This was followed by the M-PSP-1s and L-PSP-1s groups, between which no significant difference was observed (*p* > 0.05). These findings suggest that PSP-1s has the potential to ameliorate glucose intolerance in diabetic mice to a certain extent. Similar findings were also observed in the study by Lin et al. [29], where glucose tolerance was severely impaired in diabetic mice. After intervention with *Acanthopanax trifoliatus* (L.) Merr polysaccharides, the AUC of the mice decreased significantly.

### 3.9. Effects of PSP-1s on Pancreatic Pathology in Diabetic Mice

**Figure 4:**
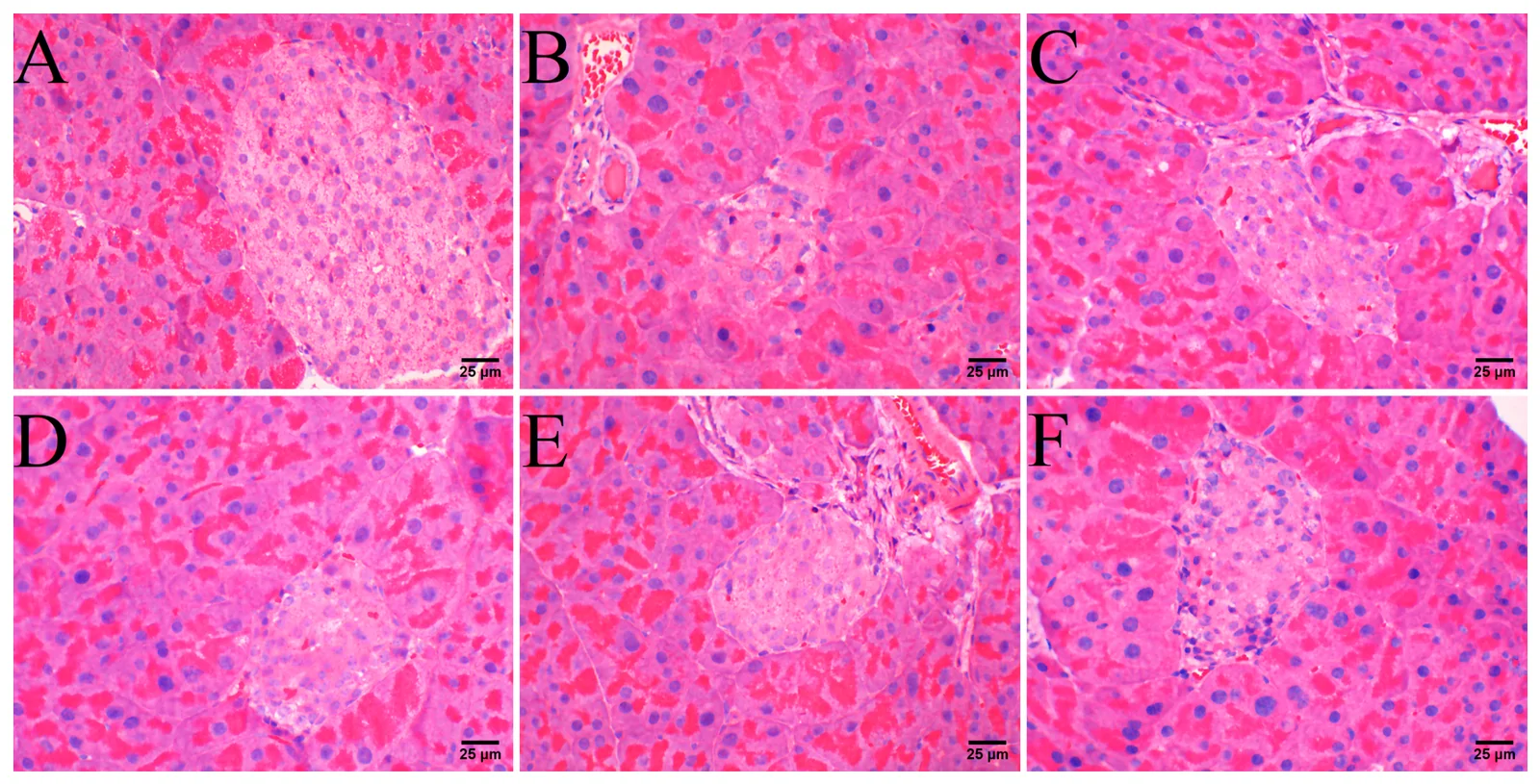
Effect of PSP-1s on pancreas of diabetic mice: (**A**) the Con group; (**B**) the Model group; (**C**) the Met group; (**D**) the L-PSP-1s group; (**E**) the M-PSP-1s group; (**F**) the H-PSP-1s group. The image was captured at a magnification of ×400.

As illustrated in Figure 4, pancreatic islets in the Con group showed clearly defined boundaries and relatively uniform cellular organization. Compared with the Model group, the representative sections from mice receiving metformin or PSP-1s intervention appeared to show less pronounced islet atrophy and clearer islet boundaries. These observations may indicate an improvement in pancreatic islet morphology following the intervention. Consistent with the findings reported by Tong et al. [56], the observed morphological changes may reflect a protective or ameliorative effect on pancreatic islets. However, these observations were based on qualitative assessment without blinded histomorphometric quantification. Further quantitative studies are needed to substantiate the apparent morphological improvements and assess their implications for islet repair or regeneration.

### 3.10. Effects of PSP-1s on Serum Biochemical in Diabetic Mice

**Figure 5:**
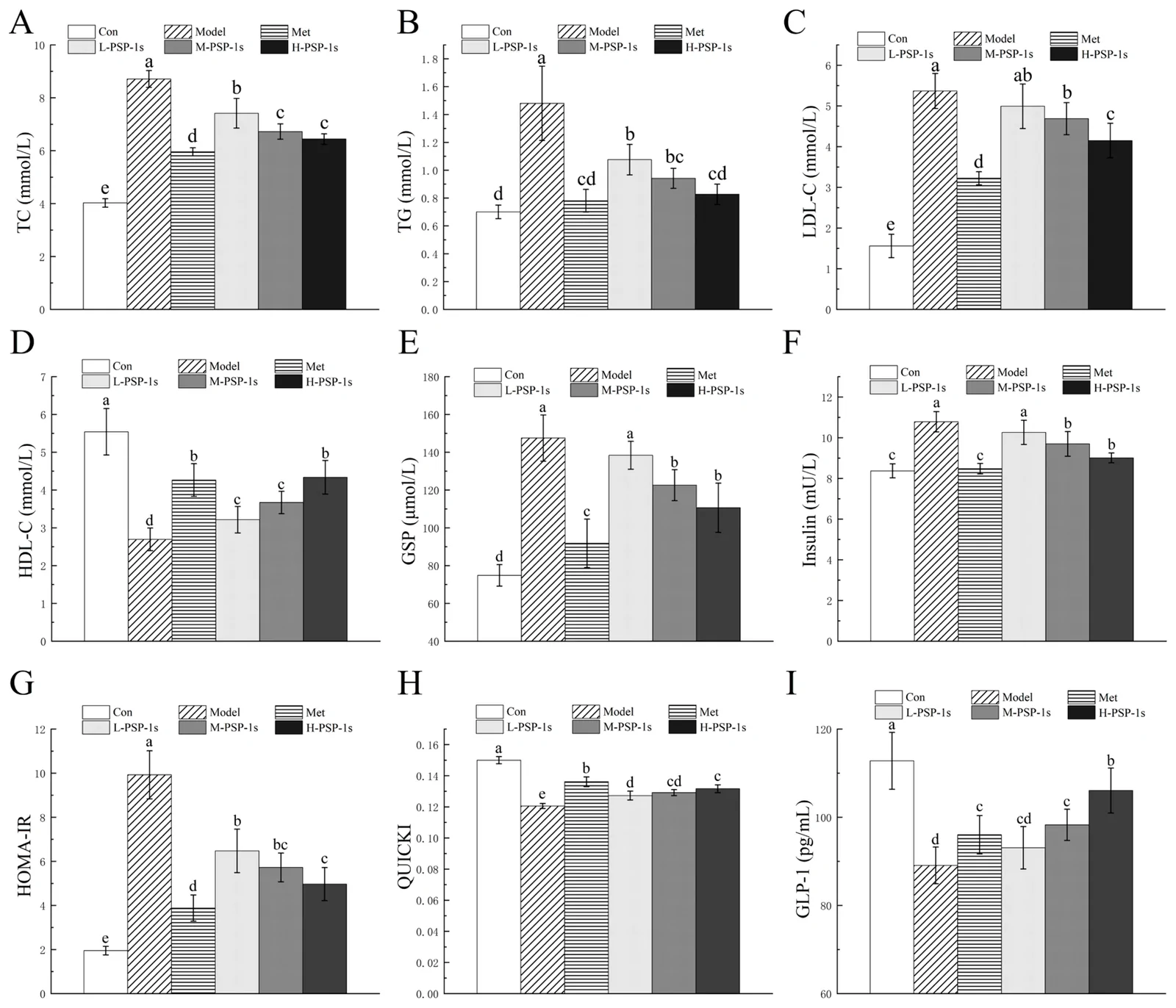
Effect of PSP-1s on serum biochemical parameters of diabetic mice: (**A**) TC; (**B**) TG; (**C**) LDL-C; (**D**) HDL-C; (**E**) GSP; (**F**) insulin; (**G**) HOMA-IR; (**H**) QUICKI; (**I**) GLP-1. Different letters represent significant differences in all groups (*p* < 0.05). *n* = 6.

The study by Diamond et al. [57] indicated that hyperglycemia is closely associated with dyslipidemia, and that type 2 diabetes complications are frequently accompanied by abnormal lipid profiles. As depicted in Figure 5A–D, the serum levels of TC, TG, and LDL-C were significantly elevated in type 2 diabetic mice compared to the Con group, while the HDL-C level was significantly reduced (*p* < 0.05). These findings suggest that the diabetic mice exhibited pronounced lipid metabolism abnormalities. The experimental data demonstrated that PSP-1s ameliorated the abnormalities in the four serum lipid markers in diabetic mice, with the ameliorative effect becoming more pronounced with increasing concentrations of PSP-1s. Specifically, in the H-PSP-1s group, the serum levels of TC, TG, and LDL-C were 26.06%, 44.12%, and 22.71% lower, respectively, than those in the Model group mice (*p* < 0.05). The HDL-C level in the H-PSP-1s group was 60.93% higher than that in the Model group mice, and the improvement effects on TG and HDL-C were comparable to those of 100 mg/kg metformin (*p* > 0.05). This improvement may result from better glucose use and reduced lipolysis, leading to lower free fatty acid production and higher fatty acid absorption in tissues [58].

GSP levels provide an assessment of average blood glucose concentrations over the preceding 2 to 3 weeks, making them a reliable marker for short-term glycemic monitoring [59]. As shown in Figure 5E, the GSP level in the Model group mice was 150.74% higher than that in the Con group (*p* < 0.05). The GSP levels in the Met group, M-PSP-1s group, and H-PSP-1s group were significantly reduced in comparison to the Model group (*p* < 0.05). The GSP level in mice of the H-PSP-1s group was 25.02% lower than that in the model group.

In type 2 diabetes, the body exhibits insulin resistance, resulting in a compensatory hypersecretion of insulin [60]. As shown in Figure 5F–H, the serum insulin level in the Model group mice was significantly higher than that in the Con group, indicating enhanced insulin resistance and reduced insulin sensitivity (*p* < 0.05). The serum insulin levels in the Met group, M-PSP-1s group, and H-PSP-1s group mice were significantly lower (*p* < 0.05). In addition, the HOMA-IR in the H-PSP-1s group was 49.95% lower than that in the Model group (*p* < 0.05), and the QUICKI was 9.17% higher (*p* < 0.05). The administration of 200 mg/kg PSP-1s resulted in a reduction of HOMA-IR that was 81.98% as effective as the reduction achieved with 100 mg/kg metformin, and an increase in QUICKI that was 71.19% as effective as that achieved with 100 mg/kg metformin. Similar to the study by Song et al. [61], which discovered that after 4 weeks of intervention with *Artemisia argyi* polysaccharide in diabetic mice, insulin levels decreased and the QUICKI improved in a dose-dependent manner, the PSP-1s in our present study also exhibited dose-dependent effects in reducing insulin levels and the Homeostatic Model Assessment of HOMA-IR, while enhancing QUICKI.

GLP-1, a pivotal incretin hormone, is essential for the regulation of glucose homeostasis and the preservation of pancreatic β-cell functionality [62]. As a significant mediator of the gut–brain axis, GLP-1 influences this axis through the hypothalamic appetite regulation center, thereby exerting further advantageous effects on blood glucose management by enhancing satiety and suppressing appetite [63]. In diabetic murine models, intestinal dysbiosis and diminished production of SCFAs impede the secretion of GLP-1 from intestinal L cells. Additionally, the cytotoxic effects of STZ on β-cells exacerbate metabolic disturbances, resulting in a further decline in GLP-1 secretion [64]. As illustrated in Figure 5I, the GLP-1 concentration in the Model group mice was reduced by 21.02% compared to the Con group. The GLP-1 level in mice from the H-PSP-1s group was 106.06 pg/mL, which was 16.01% higher than that in mice from the Model group and 14.21% higher than that in mice from the Met group (*p* < 0.05). Comparable findings were documented in the studies conducted by Wang et al. [26] and Yang et al. [1], demonstrating that polysaccharides exhibited greater efficacy than metformin in enhancing GLP-1 secretion. The results suggest that PSP-1s was associated with increased GLP-1 secretion in diabetic mice and that the serum GLP-1 level increased with increasing PSP-1s concentration, indicating that PSP-1s may be involved in the regulation of GLP-1 secretion.

### 3.11. Effects of PSP-1s on SCFAs Level in the Colonic Contents of Diabetic Mice

SCFAs are the terminal products of polysaccharide metabolism by the gut microbiota, serving as chemical mediators that facilitate communication between the microbiota and the host [65,66]. As modulators of food intake and energy metabolism, an elevation in SCFA levels triggers the release of the downstream signaling molecule GLP-1, thereby exerting an appetite-suppressing effect [15]. More precisely, SCFAs activate the cyclic adenosine monophosphate (cAMP) signaling pathway by targeting G protein-coupled receptors (GPR41 and GPR43) located on L cells, which in turn promotes the transcription and expression of the GLP-1 gene. This process results in enhanced synthesis and secretion of GLP-1 in the intestine, ultimately improving metabolism via the gut–brain axis [67].

Based on the findings discussed above, PSP-1s at a concentration of 200 mg/kg demonstrated significantly enhanced hypoglycemic activity relative to the other doses assessed. Therefore, the 200 mg/kg dosage was chosen for further exploration of PSP-1s’s effects in subsequent studies. As shown in Table 2, compared with the Con group, the contents of acetic acid, propionic acid, butyric acid, valeric acid, and isovaleric acid in mice of the Model group all decreased significantly. This indicates that diabetes induced by a high-glucose/high-fat diet combined with STZ reduces the levels of SCFAs in the intestinal tract of mice. Intervention with 200 mg/kg PSP-1s increased the contents of acetic acid, propionic acid, butyric acid, valeric acid, and isovaleric acid in the colon contents of diabetic mice (*p* < 0.05), and the effect showed no significant difference from that of 100 mg/kg metformin (*p* > 0.05). Similar findings have been reported, such as the study by Zhao et al. [68], which revealed that patients with diabetes exhibited lower levels of total SCFAs in their feces, and following intervention with *Scrophularia ningpoensis* polysaccharide, the levels of acetic acid, propionic acid, butyric acid, isobutyric acid, and valeric acid increased.

Acetic acid and propionic acid are SCFAs with relatively high contents in the intestinal tract of mice. Acetic acid serves as a crucial signaling molecule in glucose metabolism, capable of improving glucose tolerance and inhibiting weight gain in diabetic and obese rats [69]. Propionic acid participates in the gluconeogenesis pathway, suppresses the production of fat and cholesterol, increases postprandial GLP-1 levels, and enhances insulin sensitivity [10]. Under PSP-1s intervention, the contents of acetic acid and propionic acid in the colon contents of mice increased by 87.32% and 57.14% respectively compared with those in mice of the Model group. The results indicate that PSP-1s can restore the content of intestinal SCFAs.

### 3.12. Effects of PSP-1s on the Gut Microbiota in Diabetic Mice

The gut microbiota modulates host energy metabolism and feeding behavior. A growing consensus in the scientific community recognizes these microbial communities as critical determinants of both health and disease states. For example, Yao et al. [70] demonstrated that *Cyclocarya paliurus* polysaccharides alleviate type 2 diabetes symptoms in rats by lowering blood glucose and insulin levels and modifying gut microbiota. The proposed mechanism involves an increase in SCFAs-producing bacteria, which enhances the production of SCFAs.

**Figure 6:**
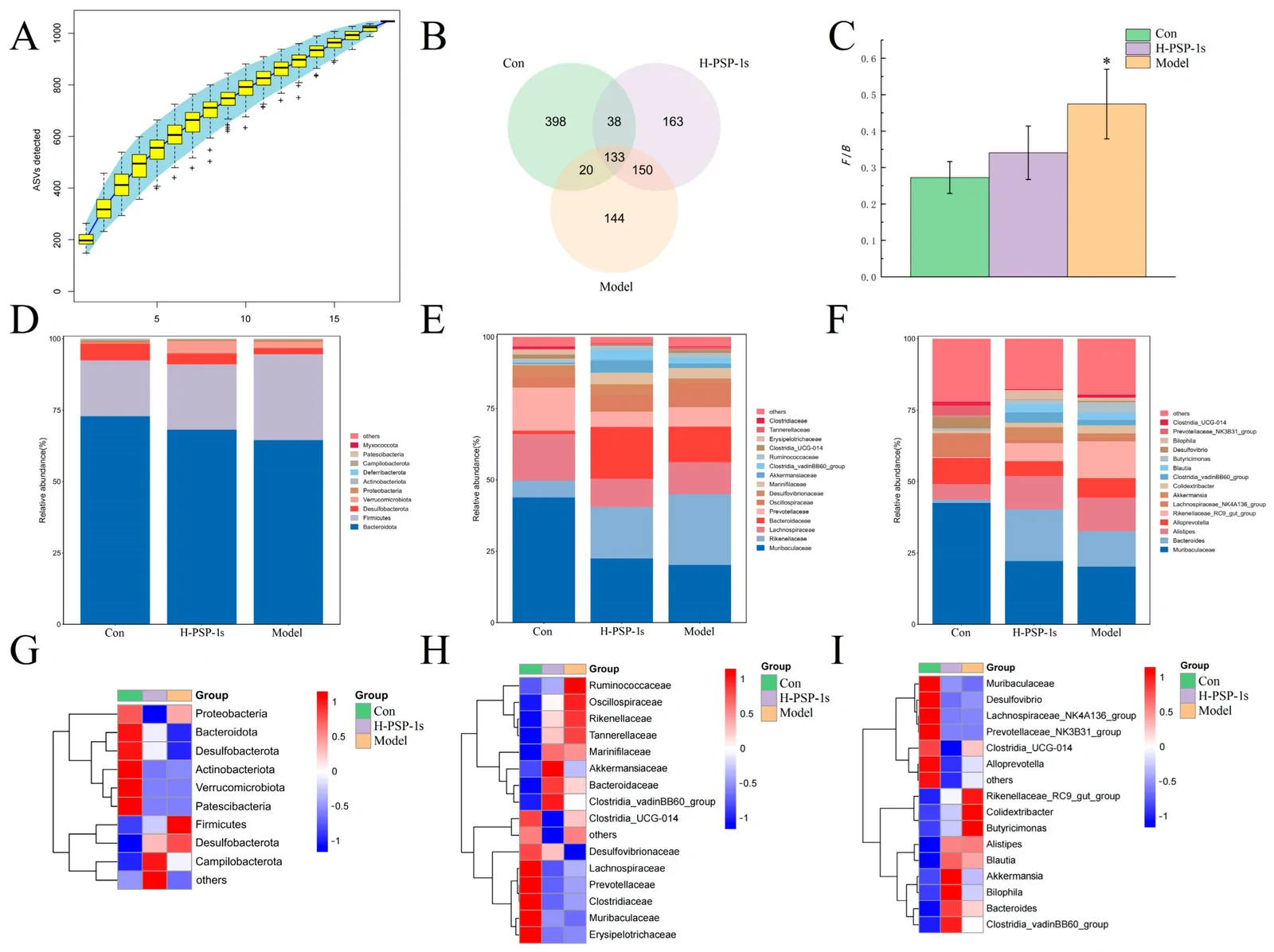
Effects of PSP-1s on the gut microbiota in diabetic mice: (**A**) species accumulation curve; (**B**) Venn diagram; (**C**) *Firmicutes*/*Bacteroides* ratio; (**D**) phylum-level species composition (Histogram); (**E**) family-level species composition (Histogram); (**F**) genus-level species composition (Histogram); (**G**) phylum-level species composition (Heatmap); (**H**) family-level species composition (Heatmap); (**I**) genus-level species composition (Heatmap). * represents significant correlations vs. the Con group: * *p* < 0.05. *n* = 6.

From Figure 6A, it can be seen that as the sample size increases, the species accumulation curve gradually flattens out, indicating that the total number of ASVs in the microbiota will no longer increase significantly with the addition of new samples. This suggests that the sample size reflects the richness of the microbial community. As shown in Figure 6B, a total of 1046 ASVs were detected in the gut microbiota of mice in the Con group, Model group, and H-PSP-1s group. Among them, 398, 144, and 163 ASVs were unique to the Con group, Model group, and H-PSP-1s group, respectively. The Model group had the fewest unique ASVs and the lowest total number of ASVs, suggesting that the composition and diversity of its gut microbiota were disrupted. This phenomenon may be attributed to elevated blood glucose levels, which stimulate the cells of the intestinal epithelium to produce substantial quantities of serotonin, thereby disrupting the gut microbiota [71]. In addition, there were 153 ASVs shared between the Con group and the Model group, 171 ASVs shared between the Con group and the H-PSP-1s group, and 283 ASVs shared between the Model group and the H-PSP-1s group. The results showed that although the gut microbiota of the three groups of mice shared a common microbial community, each group still had its own unique microbial characteristics, and PSP-1s intervention might have promoted the formation of characteristic gut microbiota while increasing microbial diversity.

As shown in Figure 6D,G, the dominant phyla in the gut microbiota of mice in each group were *Bacteroidota*, *Firmicutes*, *Desulfobacterota*, *Verrucomicrobiota*, *Proteobacteria*, *Actinobacteriota*, *Deferribacterota*, etc. Among them, *Firmicutes* and *Bacteroidota* were the dominant phyla, with a relative abundance of >85%. Diabetes can disrupt the balance of these two phyla in the gut microbiota, leading to an increase in the *Firmicutes*/*Bacteroidota* (*F*/*B*) ratio [72]. As shown in Figure 6C, the *F*/*B* ratio in the Model group was significantly higher than that in the Con group (*p* < 0.05). After PSP-1s intervention, the *F*/*B* ratio in diabetic mice significantly decreased compared to the Model group (*p* < 0.05), an effect also observed with *Lycium barbarum* polysaccharide [14], which exhibits hypoglycemic activity. In addition, the abundance of *Desulfobacterota* in the gut microbiota of the Model group was lower than that in the Con group, while the abundance of *Desulfobacterota* in the gut microbiota of the H-PSP-1s group was higher than that in the Model group. The H-PSP-1s group had an increased abundance of *Verrucomicrobiota* compared to both the Con group and the Model group. Similar findings were reported in the study by Hu et al. [73], which demonstrated that extracellular polysaccharides produced by *Sporidiobolus pararoseus* could enhance the relative abundance of *Desulfobacterota* and *Verrucomicrobiota*, promote the proliferation of beneficial bacteria in the human and mouse gut, and stimulate the production of SCFAs and amino acid metabolism, thereby improving intestinal health.

As shown in Figure 6E,H, the dominant families in the gut microbiota of mice in each group were *Muribaculaceae*, *Rikenellaceae*, *Lachnospiraceae*, *Bacteroidaceae*, *Prevotellaceae*, *Oscillospiraceae*, *Desulfovibrionaceae*, *Marinifilaceae*, *Akkermansiaceae*, *Ruminococcaceae*, etc. The Model group had increased abundances of *Rikenellaceae*, *Oscillospiraceae*, and *Ruminococcaceae* in the gut microbiota compared to the Con group, while the H-PSP-1s group had decreased abundances of these bacteria compared to the Model group. The *Muribaculaceae* family is recognized as a beneficial group of bacteria that contributes to maintaining intestinal homeostasis, modulating the composition of gut microbiota, and enhancing the secretion of SCFAs in diabetic mice [74]. Song et al. [61] identified that *Artemisia argyi* polysaccharide can increase the abundance of *Muribaculaceae*. In the present study, the abundance of *Muribaculaceae* in the Model group mice was lower than that in the Con group mice, and PSP-1s intervention led to an upregulation of *Muribaculaceae* abundance.

As shown in Figure 6F,I, at the genus level, the dominant bacterial genera in the intestinal tracts of mice across different groups are *Muribaculaceae*, *Bacteroides*, *Alistipes*, *Alloprevotella*, *Rikenellaceae RC9 gut group*, *Lachnospiraceae NK4A136 group*, *Akkermansia*, *Colidextribacter*, *Clostridia vadinBB60 group*, *Blautia*, etc. In comparison to the Model group, the abundance of *Muribaculaceae* increased in mice following PSP-1s intervention. Furthermore, relative to both the Con and Model groups, the abundances of *Bacteroides*, *Akkermansia*, *Clostridia vadinBB60 group*, and *Blautia* in the gut microbiota of mice treated with PSP-1s were elevated. Previous research has indicated that *Muribaculaceae* [75], *Bacteroides* [76], *Akkermansia* [77], and *Blautia* [78] are capable of producing SCFAs. Notably, *Akkermansia muciniphila*, a species within the *Akkermansia* genus, has attracted significant attention in recent studies focused on mitigating the risks associated with diabetes and obesity. Evidence suggests that this bacterium can enhance glucose homeostasis in mice and reduce the risks of conditions such as diabetes, obesity, inflammatory bowel disease, and colorectal cancer. Additionally, a novel protein (P9) secreted by *Akkermansia muciniphila* has been shown to stimulate the secretion of GLP-1 [79,80].

**Figure 7:**
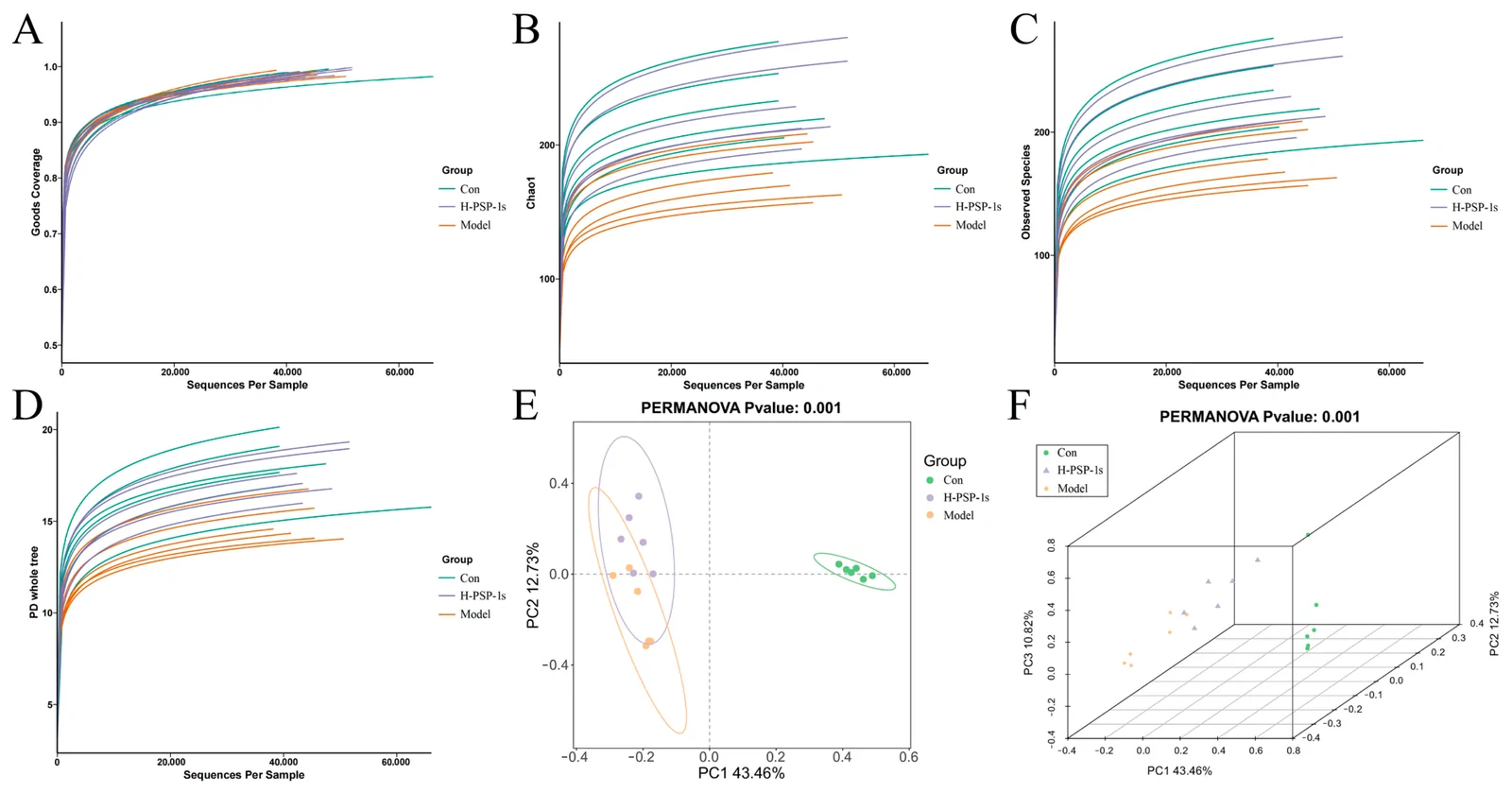
Analysis of α- and β-diversity of the gut microbiota in diabetic mice: (**A**) Goods Coverage; (**B**) Chao1; (**C**) Observed Species; (**D**) PD whole tree; (**E**) PCoA based on binary Jaccard distance (2D) analysis; (**F**) PCoA based on binary Jaccard distance (3D) analysis. *n* = 6.

The results of the α-diversity analysis are shown in Figure 7A–D and Table S8. The Good’s Coverage index of all groups was close to 1, indicating a high reliability of the sequencing results. The Chao1, Observed Species, and ACE indices in the Model group were significantly lower than those in the Con group and the H-PSP-1s group (*p* < 0.05), while there was no significant difference between the Con group and the H-PSP-1s group (*p* > 0.05). This suggests that the richness of the gut microbiota in the Model group was reduced, and PSP-1s intervention could restore the richness of the gut microbiota. The results of the β-diversity analysis are shown in Figure 7E,F. The PCoA results showed that the microbial community compositions of samples within each group were similar, whereas differences were observed between groups. Permutational multivariate analysis of variance (PERMANOVA) was used to statistically confirm these differences, and the results indicated significant differences among groups (*p* = 0.001). These findings suggest that PSP-1s not only alleviated the abnormal changes in the gut microbiota of diabetic mice but also promoted the formation of a microbial pattern distinct from those of the Con and Model groups.

**Figure 8:**
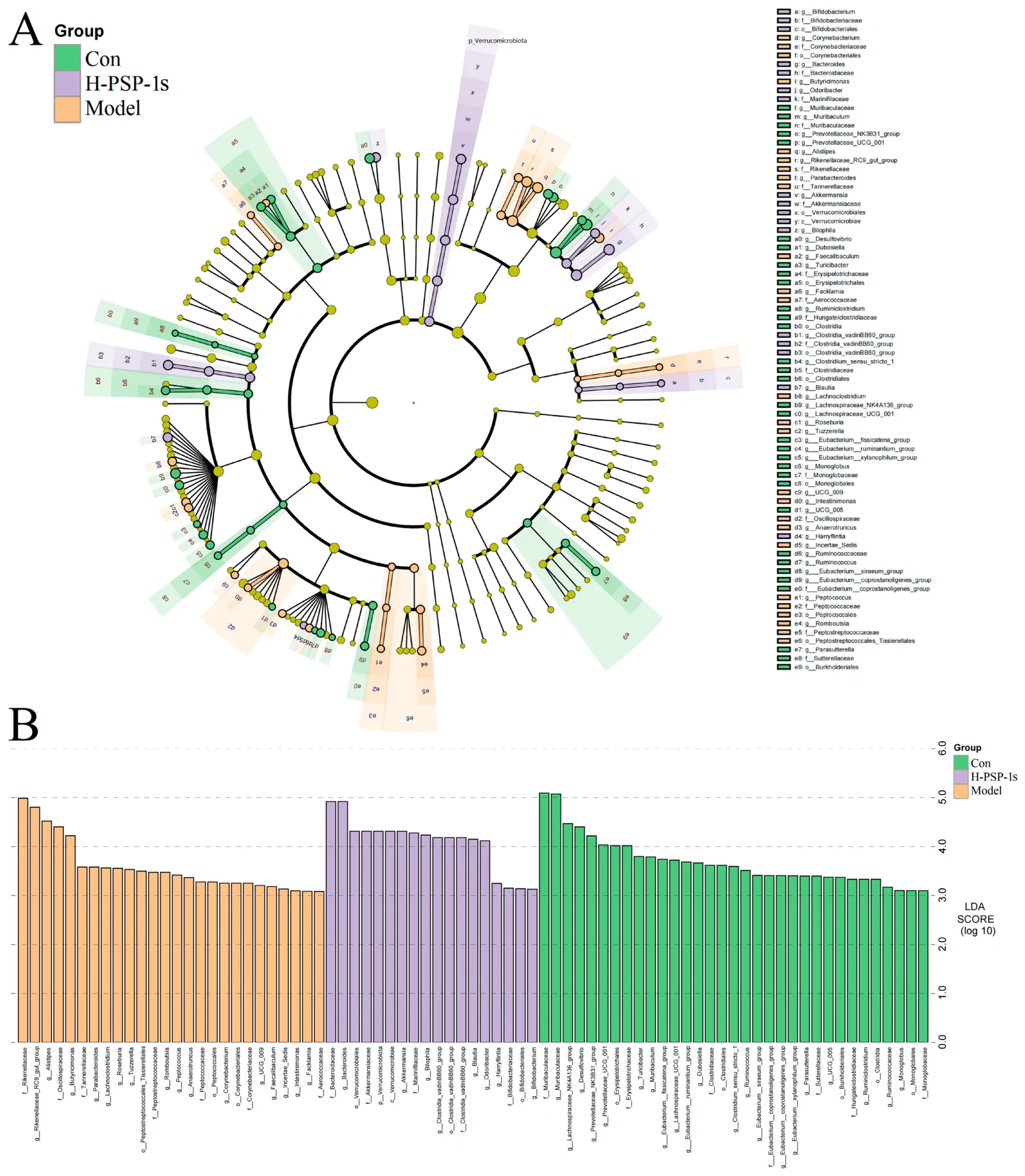
LEfSe analysis results of the gut microbiota in diabetic mice: (**A**) differential species annotation cladogram; (**B**) LDA value distribution bar chart. *n* = 6.

Utilizing linear discriminant analysis (LDA) effect size (LEfSe) analysis (LDA > 3, *p* < 0.05), we identified distinct species exhibiting statistically significant differences across groups. The results are shown in Figure 8. Among the differential species in the gut microbiota of the H-PSP-1s group, *Bacteroidaceae*, *Bacteroides* [76], *Verrucomicrobiae*, *Verrucomicrobiales*, *Akkermansiaceae*, *Akkermansia* [77], *Blautia* [78], *Odoribacter* [81], *Bifidobacteriales*, *Bifidobacteriaceae*, and *Bifidobacterium* [82] have all been reported as beneficial bacteria capable of producing SCFAs. The *Clostridia vadinBB60 group* also belongs to beneficial bacteria [83]. In a human study, Sanmiguel et al. [84] found that *Akkermansia* was significantly associated with a reduction in hedonic eating, while *Bilophila* showed a significant association with weight loss. The above results indicate that PSP-1s promotes the increase of beneficial bacteria, especially those microbial communities capable of producing SCFAs. Some polysaccharides with hypoglycemic effects exhibit similar outcomes. For instance, the study by Pan et al. [85] demonstrated that *Morchella importuna* polysaccharide can reverse intestinal diseases caused by type 2 diabetes by increasing the abundances of *Akkermansia* and *Blautia*, and the research by Ni et al. [86] showed that *Ramaria botrytoides* polysaccharide can improve the gut microbiota of diabetic mice and also increase the abundances of *Bacteroides*, *Odoribacter*, and *Akkermansia*.

### 3.13. Effects of PSP-1s on Protein Expression in the Hypothalamus of Diabetic Mice

**Figure 9:**
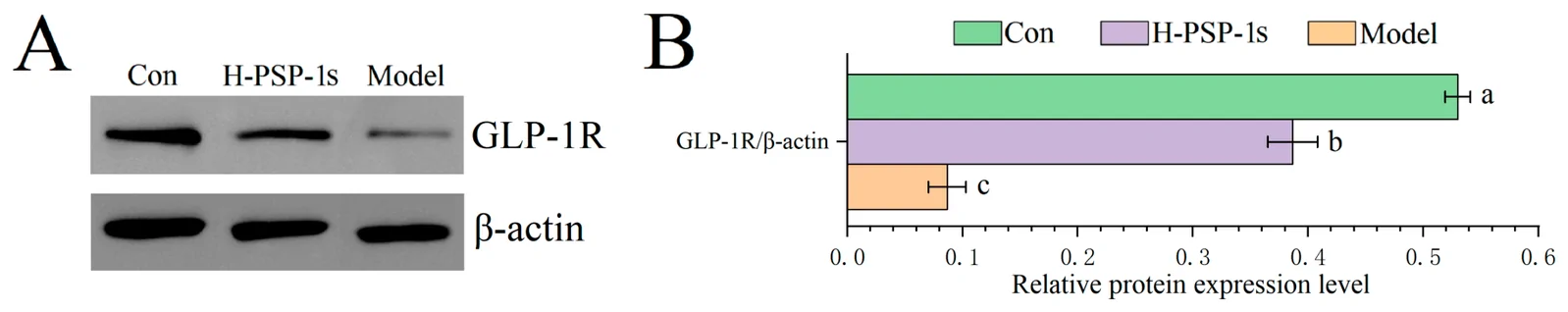
Effect of PSP-1s on GLP-1R protein expression in the hypothalamus of diabetic mice: (**A**) Representative WB bands; (**B**) the bar graph of the relative intensities of WB bands. Different letters represent significant differences in all groups (*p* < 0.01). *n* = 6.

GLP-1 exerts its effects on the GLP-1R located within the arcuate nucleus (ARC) of the hypothalamus, a region recognized as a critical center for the regulation of feeding behavior [87]. Activation of the GLP-1R facilitates alterations in feeding behavior and stimulates insulin secretion, thereby influencing abnormal blood glucose fluctuations associated with dietary intake [88]. Prior studies have demonstrated that the GLP-1/GLP-1R system within the brain–gut axis plays a mediating and beneficial role in maintaining glucose homeostasis in patients with type 2 diabetes [89]. The study by Yang et al. [1] found that Finger citron polysaccharide could increase the serum GLP-1 level in diabetic mice and activate the expression of GLP-1R in the hypothalamus. As shown in Figure 9, GLP-1R protein expression in the hypothalamus of Model group mice was significantly reduced to 16.35% of the level in the Con group (*p* < 0.01). After intervention with PSP-1s, the protein expression level of GLP-1R in the hypothalamus was significantly increased (*p* < 0.01), reaching 72.96% of that in the Con group. The increased hypothalamic GLP-1R protein expression in the H-PSP-1s group, together with the observed reduction in food intake, suggests that hypothalamic GLP-1 signaling may be involved in the response to PSP-1s intervention [90].

### 3.14. Effects of PSP-1s on Gene Expression in the Hypothalamus of Diabetic Mice

POMC/CART neurons and NPY/AgRP neurons are two independent neuronal populations in the ARC of the hypothalamus, playing a pivotal role in regulating appetite [87]. Two of these genes, POMC and CART, are anorexigenic neuropeptides whereas the other two, NPY and AgRP, are orexigenic [22]. For instance, saponins are believed to suppress appetite and subsequently reduce energy intake by regulating POMC/CART neurons and NPY/AgRP neurons in the hypothalamus, the appetite control center [91]. The research conducted by Li et al. [87] revealed that the administration of highly expanded konjac fiber and konjac powder resulted in increased plasma GLP-1 levels in rats, accompanied by enhanced protein expression of GLP-1R in the hypothalamus. Simultaneously, there was a downregulation of NPY and AgRP gene expression in the hypothalamus, which collectively contributed to a decrease in food consumption and mitigated weight gain. Nevertheless, there is a lack of reported studies examining polysaccharides in a similar context.

**Figure 10:**
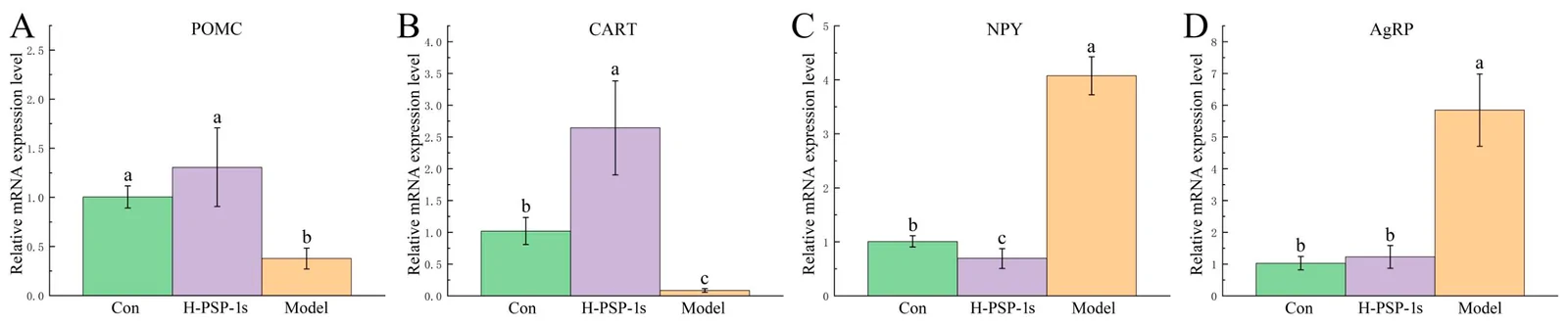
Effect of PSP-1s on appetite-related gene expression in the hypothalamus of diabetic mice: (**A**) POMC; (**B**) CART; (**C**) NPY; (**D**) AgRP. Different letters represent significant differences in all groups (*p* < 0.05). *n* = 6.

As shown in Figure 10, in the hypothalamus of mice in the H-PSP-1s group, the expression level of the POMC gene was significantly increased by 241.70% compared to that in the Model group mice (*p* < 0.05). Compared with both the Con group and the Model group mice, the expression level of the CART gene was significantly increased by 159.61% and 3020.35%, respectively (*p* < 0.05). This indicates that PSP-1s enhances the expression of appetite suppression-related genes in the hypothalamus. Conversely, in the hypothalamus of mice in the H-PSP-1s group, the expression level of the NPY gene was significantly decreased by 30.93% and 82.99%, respectively, compared to the Con group and the Model group mice (*p* < 0.05). The expression level of the AgRP gene was significantly decreased by 79.04% compared to the Model group mice (*p* < 0.05). This suggests that PSP-1s inhibits the expression of appetite-stimulating genes in the hypothalamus. These changes in hypothalamic appetite-related gene expression were consistent with the reduced food intake observed following PSP-1s treatment, suggesting a possible involvement of hypothalamic appetite regulation. However, the causal relationship between these transcriptional changes and feeding behavior remains to be established.

## 4. Conclusions

This study revealed that the three pumpkin seed polysaccharide fractions, PSP-1-1, PSP-1-2, and PSP-1-3, had weight-average molecular weights of 29,797 Da, 11,209 Da, and 4346 Da, respectively, and all were likely to contain both α- and β-glycosidic linkages. PSP-1-1 was composed of 11 types of glycosidic linkages, with arabinose, galactose, and glucose as the predominant sugar residues. Both PSP-1-2 and PSP-1-3 consisted of 14 types of glycosidic linkages, with arabinose, xylose, galactose, and glucose as the main sugar residues. All three fractions shared common structural units, including Araf-(1→, Glcp-(1→ as terminal residues, as well as →5)-Araf-(1→ and →3)-Galp-(1→ linkages.

In NCI-H716 cells, pumpkin seed polysaccharide fractions increased GLP-1 secretion and upregulated the mRNA expression of T1R2, T1R3, α-gustducin, PLCβ2, IP3R, and TRPM5. PSP-1-1 and PSP-1s also reduced GLUT2 and SGLT1 mRNA expression. These findings suggest a possible involvement of sweet taste receptor-related signaling, but do not establish receptor activation or pathway dependence. The GLP-1 secretion-promoting activity and gene expression responses of the pooled fraction PSP-1s supported its selection for subsequent animal experiments. However, synergistic interactions among its constituent fractions were not established.

In diabetic mice, PSP-1s intervention alleviated weight loss, reduced food and water intake, lowered fasting blood glucose, improved glucose tolerance and dyslipidemia, and decreased GSP, insulin, and HOMA-IR while increasing QUICKI. Qualitative histological observations suggested an improvement in pancreatic islet morphology following the intervention. These effects were accompanied by increased serum GLP-1 and colonic SCFA concentrations, altered gut microbiota composition, increased hypothalamic GLP-1R protein expression, and changes in appetite-related gene expression.

Taken together, the findings suggest that the hypoglycemic effects of PSP-1s may involve GLP-1-related regulation through intestinal and hypothalamic responses. The cellular results point to a possible involvement of sweet taste receptor-related signaling, while the animal findings suggest potential links among gut microbiota composition, SCFAs, GLP-1, and hypothalamic appetite regulation. These observations provide a basis for investigating gut–brain signaling as a potential mechanism underlying the effects of PSP-1s, although the causal relationships among these changes require further functional validation. The pancreatic histological findings remain qualitative, and the long-term safety of PSP-1s also warrants further investigation.

## Figures and Tables

**Table 1:** Comparison of the diets of mice in the Con and HFD groups.

Nutrient	Con Group (kcal%)	HFD Group (kcal%)
Protein	20.6	20
Carbohydrate	67.4	20
Fat	12	60
Total (kcal/kg)	2530	5240

**Table 2:** Content of SCFAs in the colon contents of diabetic mice. Different letters represent significant differences in all groups (*p* < 0.05). *n* = 6.

SCFAs (μg/mg)	Con	Model	Met	H-PSP-1s
Acetic acid	1.71 ± 0.29 ^a^	0.71 ± 0.10 ^c^	1.13 ± 0.13 ^b^	1.33 ± 0.18 ^b^
Propionic acid	0.42 ± 0.06 ^a^	0.14 ± 0.03 ^c^	0.21 ± 0.02 ^b^	0.22 ± 0.02 ^b^
Butyric acid	0.25 ± 0.03 ^a^	0.18 ± 0.02 ^c^	0.21 ± 0.04 ^bc^	0.23 ± 0.03 ^ab^
Isobutyric acid	0.05 ± 0.01 ^a^	0.04 ± 0.01 ^a^	0.05 ± 0.01 ^a^	0.05 ± 0.01 ^a^
Valeric acid	0.12 ± 0.01 ^a^	0.10 ± 0.01 ^c^	0.12 ± 0.01 ^ab^	0.11 ± 0.01 ^b^
Isovaleric acid	0.07 ± 0.01 ^a^	0.05 ± 0.01 ^c^	0.06 ± 0.01 ^bc^	0.06 ± 0.01 ^ab^

## Data Availability

The data presented in this study are available on request from the corresponding author since the data are part of an ongoing study.

## Supplementary Materials

The following supporting information can be downloaded at: https://www.mdpi.com/article/10.3390/foods15213788/s1. Table S1: The physicochemical properties and yields of each pumpkin seed polysaccharide fraction; Table S2: RT-qPCR primer design for determination of sweet taste receptor signaling pathway-related gene expression in NCI-H716 cells; Table S3: SCFAs reference standards calibration curve; Table S4: RT-qPCR primer design for determination of appetite-related gene expression in mice hypothalamus; Table S5: Glycosyl linkages analysis of PSP-1-1; Table S6: Glycosyl linkages analysis of PSP-1-2; Table S7: Glycosyl linkages analysis of PSP-1-3; Table S8: α-diversity analysis of the gut microbiota in diabetic mice; Figure S1: Isolation and Purification of Pumpkin Seed Polysaccharides: (A) DEAE-52 cellulose column elution curve and (B) Sephadex-75 gel column elution curve.

## References

[1] Yang Y., Luo J., Wang S., Yang D., Chen S., Wang Q., Zhou A. (2025). A water-soluble polysaccharide from finger citron ameliorates diabetes mellitus via gut microbiota-GLP-1 pathway in high-fat diet and streptozotocin-induced diabetic mice. Int. J. Biol. Macromol..

[2] Xia T., Liu C.S., Hu Y.N., Luo Z.Y., Chen F.L., Yuan L.X., Tan X.M. (2021). Coix seed polysaccharides alleviate type 2 diabetes mellitus via gut microbiota-derived short-chain fatty acids activation of IGF1/PI3K/AKT signaling. Food Res. Int..

[3] Shao W., Xiao C., Yong T., Zhang Y., Hu H., Xie T., Liu R., Huang L., Li X., Xie Y. (2022). A polysaccharide isolated from Ganoderma lucidum ameliorates hyperglycemia through modulating gut microbiota in type 2 diabetic mice. Int. J. Biol. Macromol..

[4] Xie S.Z., Yang G., Jiang X.M., Qin D.Y., Li Q.M., Zha X.Q., Pan L.H., Jin C.S., Luo J.P. (2020). Polygonatum cyrtonema Hua Polysaccharide Promotes GLP-1 Secretion from Enteroendocrine L-Cells through Sweet Taste Receptor-Mediated cAMP Signaling. J. Agric. Food Chem..

[5] Cai S., Zhong C., Li M., Luo J., Zhou A. (2025). Isolation, Purification, and In Vitro Hypoglycemic Activity of Pumpkin Seed Polysaccharides. Sci. Technol. Food Ind..

[6] Fatima H., Hussain A., Ambreen, Kabir K., Arshad F., Ayesha A., Bibi B., Ahmed A., Najam A., Firdous N. (2025). Pumpkin seeds; an alternate and sustainable source of bioactive compounds and nutritional food formulations. J. Food Compos. Anal..

[7] Makki K., Deehan E.C., Walter J., Bäckhed F. (2018). The Impact of Dietary Fiber on Gut Microbiota in Host Health and Disease. Cell Host Microbe.

[8] Feng X., Wang D., Hu L., Lu H., Ling B., Huang Y., Jiang Q. (2022). Dendrobium officinale polysaccharide ameliorates polycystic ovary syndrome via regulating butyrate dependent gut-brain-ovary axis mechanism. Front. Endocrinol..

[9] Luo J., Li Y., Mai Y., Gao L., Ou S., Wang Y., Liu L., Peng X. (2018). Flaxseed gum reduces body weight by regulating gut microbiota. J. Funct. Foods.

[10] Chambers E.S., Viardot A., Psichas A., Morrison D.J., Murphy K.G., Zac-Varghese S.E., MacDougall K., Preston T., Tedford C., Finlayson G.S. (2015). Effects of targeted delivery of propionate to the human colon on appetite regulation, body weight maintenance and adiposity in overweight adults. Gut.

[11] Chakraborty S., Beura M., Sharma S.K., Singh A., Dahuja A., Krishnan V. (2023). Lentinan, β-glucan from Shiitake (*Lentinula edodes*): A review on structure, conformational transition, and gastro-intestinal interaction contributing towards its anti-diabetic potential. Trends Food Sci. Technol..

[12] Chen S.-M., Zeng F.-S., Fu W.-W., You H.-T., Mu X.-Y., Chen G.-F., Lv H., Li W.-J., Xie M.-Y. (2023). White hyacinth bean polysaccharide ameliorates diabetes via microbiota-gut-brain axis in type 2 diabetes mellitus rats. Int. J. Biol. Macromol..

[13] Xia T., He W., Luo Z., Wang K., Tan X. (2024). Achyranthes bidentata polysaccharide ameliorates type 2 diabetes mellitus by gut microbiota-derived short-chain fatty acids-induced activation of the GLP-1/GLP-1R/cAMP/PKA/CREB/INS pathway. Int. J. Biol. Macromol..

[14] Ma Q., Zhai R., Xie X., Chen T., Zhang Z., Liu H., Nie C., Yuan X., Tu A., Tian B. (2022). Hypoglycemic Effects of Lycium barbarum Polysaccharide in Type 2 Diabetes Mellitus Mice via Modulating Gut Microbiota. Front. Nutr..

[15] Medawar E., Haange S.B., Rolle-Kampczyk U., Engelmann B., Dietrich A., Thieleking R., Wiegank C., Fries C., Horstmann A., Villringer A. (2021). Gut microbiota link dietary fiber intake and short-chain fatty acid metabolism with eating behavior. Transl. Psychiatry.

[16] Yang Z., Wang Y., Chen S. (2021). Astragalus polysaccharide alleviates type 2 diabetic rats by reversing the glucose transporters and sweet taste receptors/GLP-1/GLP-1 receptor signaling pathways in the intestine-pancreatic axis. J. Funct. Foods.

[17] Wang S., Yang Y., Wang Q., Wu Z., Liu X., Chen S., Zhou A. (2023). Structural characterization and immunomodulatory activity of a polysaccharide from finger citron extracted by continuous phase-transition extraction. Int. J. Biol. Macromol..

[18] Ji X., Zhang F., Zhang R., Liu F., Peng Q., Wang M. (2019). An acidic polysaccharide from Ziziphus Jujuba cv. Muzao: Purification and structural characterization. Food Chem..

[19] Sung W.W., Tu J.H., Yu J.S., Ulfa M.Z., Chang J.H., Cheng H.L. (2021). Bacillus amyloliquefaciens exopolysaccharide preparation induces glucagon-like peptide 1 secretion through the activation of bitter taste receptors. Int. J. Biol. Macromol..

[20] Xu Y., Carradori D., Alhouayek M., Muccioli G.G., Cani P.D., Préat V., Beloqui A. (2018). Size Effect on Lipid Nanocapsule-Mediated GLP-1 Secretion from Enteroendocrine L Cells. Mol. Pharm..

[21] Okimura T., Jiang Z., Liang Y., Yamaguchi K., Oda T. (2019). Suppressive effect of ascophyllan HS on postprandial blood sugar level through the inhibition of α-glucosidase and stimulation of glucagon-like peptide-1 (GLP-1) secretion. Int. J. Biol. Macromol..

[22] Bai F., Sözen M.A., Lukiw W.J., Argyropoulos G. (2005). Expression of AgRP, NPY, POMC and CART in human fetal and adult hippocampus. Neuropeptides.

[23] Kim K.-S., Yang H.J., Lee I.-S., Kim K.-H., Park J., Jeong H.-S., Kim Y., Ahn K.S., Na Y.-C., Jang H.-J. (2015). The aglycone of ginsenoside Rg3 enables glucagon-like peptide-1 secretion in enteroendocrine cells and alleviates hyperglycemia in type 2 diabetic mice. Sci. Rep..

[24] Wang L., Zhou Y., Chen Z., Sun L., Wu J., Li H., Liu F., Wang F., Yang C., Yang J. (2019). PLCβ2 negatively regulates the inflammatory response to virus infection by inhibiting phosphoinositide-mediated activation of TAK1. Nat. Commun..

[25] Sun W., Zhang Y., Jia L. (2022). Polysaccharides from Agrocybe cylindracea residue alleviate type 2-diabetes-induced liver and colon injuries by p38 MAPK signaling pathway. Food Biosci..

[26] Wang Y.X., Pi J.C., Yao Y.F., Peng X.P., Li W.J., Xie M.Y. (2024). Hypoglycemic effects of white hyacinth bean polysaccharide on type 2 diabetes mellitus rats involvement with entero-insular axis and GLP-1 via metabolomics study. Int. J. Biol. Macromol..

[27] Zhu J.H., Mao Q., Wang S.Y., Liu H., Zhou S.S., Zhang W., Kong M., Zhu H., Li S.L. (2022). Optimization and validation of direct gas chromatography-mass spectrometry method for simultaneous quantification of ten short-chain fatty acids in rat feces. J. Chromatogr. A.

[28] Chen Y., Liu Y., Sarker M.M.R., Yan X., Yang C., Zhao L., Lv X., Liu B., Zhao C. (2018). Structural characterization and antidiabetic potential of a novel heteropolysaccharide from Grifola frondosa via IRS1/PI3K-JNK signaling pathways. Carbohydr. Polym..

[29] Lin Y., Pan J., Liu Y., Yang H., Wu G., Pan Y. (2023). *Acanthopanax trifoliatus* (L.) Merr polysaccharides ameliorates hyperglycemia by regulating hepatic glycogen metabolism in type 2 diabetic mice. Front. Nutr..

[30] Yan S., Pan C., Yang X.Q., Chen S., Qi B., Huang H. (2021). Degradation of Codium cylindricum polysaccharides by H_2_O_2_-Vc-ultrasonic and H_2_O_2_-Fe^2+^-ultrasonic treatment: Structural characterization and antioxidant activity. Int. J. Biol. Macromol..

[31] Zhou N., Zhao Y., Zhang L., Ning Y. (2022). Protective effects of black onion polysaccharide on liver and kidney injury in T2DM rats through the synergistic impact of hypolipidemic and antioxidant abilities. Int. J. Biol. Macromol..

[32] Rozi P., Abuduwaili A., Bao X., Zhu J., Yadikar N., Wang J., Ma S., Yang X., Yili A. (2019). Isolations, characterizations and bioactivities of polysaccharides from the seeds of three species Glycyrrhiza. Int. J. Biol. Macromol..

[33] Tang Y., He X., Liu G., Wei Z., Sheng J., Sun J., Li C., Xin M., Li L., Yi P. (2023). Effects of different extraction methods on the structural, antioxidant and hypoglycemic properties of red pitaya stem polysaccharide. Food Chem..

[34] Zhong W., Yang C.X., Zhang Y., Yang D. (2022). The prebiotic properties of polysaccharides obtained by differentiated deproteinization methods from Flos Sophorae Immaturus on *Lactobacillus fermentum*. Front. Microbiol..

[35] Ozaki S., Oki N., Suzuki S., Kitamura S. (2010). Structural characterization and hypoglycemic effects of arabinogalactan-protein from the tuberous cortex of the white-skinned sweet potato (*Ipomoea batatas* L.). J. Agric. Food Chem..

[36] He J., Fang J., Yu C., Zhang X., Sun P., Ren Y. (2024). Purification, structural identification, in vitro hypoglycemic activity and digestion characteristics of polysaccharides from the flesh and peel of wampee (*Clausena lansium*). Food Res. Int..

[37] Zhang Z., Lai J., Fan X., Wang S., Zhang H., Wang L., Wang H. (2024). Extraction of polysaccharides from *Polygonum cuspidatum* with activity against Type 2 Diabetes via alterations in gut microbiota. Food Chem..

[38] Hui H., Jin H., Yang X., Wang X., Qin B. (2024). Fine structure and hypoglycemic effect of a galactoglucan from the bulbs of Lanzhou lily. Int. J. Biol. Macromol..

[39] Wang M., Zhao Z., Xue J., Hu J., Zhang J., Liu P. (2020). Hypoglycemic Activity of Purified Polysaccharides from *Inonotus obliquus* In Vitro. Sci. Technol. Food Ind..

[40] Smith K., Karimian Azari E., LaMoia T.E., Hussain T., Vargova V., Karolyi K., Veldhuis P.P., Arnoletti J.P., de la Fuente S.G., Pratley R.E. (2018). T1R2 receptor-mediated glucose sensing in the upper intestine potentiates glucose absorption through activation of local regulatory pathways. Mol. Metab..

[41] Jang H., Kokrashvili Z., Theodorakis M.J., Carlson O.D., Kim B., Zhou J., Kim H.H., Xu X., Chan S.L., Juhaszova M. (2007). Gut-Expressed Gustducin and Taste Receptors Regulate Secretion of Glucagon-Like Peptide-1. Proc. Natl. Acad. Sci. USA.

[42] Macrae C., Lalović D., Bunney T.D., Katan M. (2025). Phosphoinositide-specific phospholipase C enzymes: Recent advances in a long journey. Biochim. Biophys. Acta (BBA)-Mol. Cell Biol. Lipids.

[43] Diepeveen J., Moerdijk-Poortvliet T.C., van der Leij F.R. (2022). Molecular insights into human taste perception and umami tastants: A review. J. Food Sci..

[44] Palmer R.K. (2007). The pharmacology and signaling of bitter, sweet, and umami taste sensing. Mol. Interv..

[45] Ohtsu Y., Nakagawa Y.S., Nagasawa M., Takeda S., Arakawa H., Kojima I. (2014). Diverse signaling systems activated by the sweet taste receptor in human GLP-1-secreting cells. Mol. Cell. Endocrinol..

[46] Wang F., Song X., Zhou L., Liang G., Huang F., Jiang G., Zhang L. (2018). The downregulation of sweet taste receptor signaling in enteroendocrine L-cells mediates 3-deoxyglucosone-induced attenuation of high glucose-stimulated GLP-1 secretion. Arch. Physiol. Biochem..

[47] Koepsell H. (2020). Glucose transporters in the small intestine in health and disease. Pflügers Arch.-Eur. J. Physiol..

[48] Wemelle E., Carneiro L., Abot A., Lesage J., Cani P.D., Knauf C. (2022). Glucose Stimulates Gut Motility in Fasted and Fed Conditions: Potential Involvement of a Nitric Oxide Pathway. Nutrients.

[49] Lin X., Xu W., Liu L., Ou S., Peng X. (2021). In vitro fermentation of flaxseed polysaccharide by fecal bacteria inhibits energy intake and adipogenesis at physiological concentration. Food Res. Int..

[50] Yang H., Gong F., Liu T., Li T., Wu L. (2023). Antihyperglycemic mechanism of *Polygonatum odoratum* polysaccharides based on sweet taste receptor signaling pathway. Chin. Tradit. Pat. Med..

[51] Castillo Í.M.P., Argilés J.M., Rueda R., Ramírez M., Pedrosa J.M.L. (2025). Skeletal muscle atrophy and dysfunction in obesity and type-2 diabetes mellitus: Myocellular mechanisms involved. Rev. Endocr. Metab. Disord..

[52] Alkhalidy H., Moore W., Wang Y., Luo J., McMillan R.P., Zhen W., Zhou K., Liu D. (2018). The Flavonoid Kaempferol Ameliorates Streptozotocin-Induced Diabetes by Suppressing Hepatic Glucose Production. Molecules.

[53] Talbot S.R., Heider M., Wirth M., Jörns A., Naujok O. (2024). Exploring dose-response variability and relative severity assessment in STZ-induced diabetes male NSG mice. Sci. Rep..

[54] Wang Z., Wang Z., Huang W., Suo J., Chen X., Ding K., Sun Q., Zhang H. (2020). Antioxidant and anti-inflammatory activities of an anti-diabetic polysaccharide extracted from *Gynostemma pentaphyllum* herb. Int. J. Biol. Macromol..

[55] Gileles-Hillel A., Soesman L.H., Miari S., Breuer O., Tsabari R., Elyashar-Earon H., Armoni S., Sprikkelman A.B., Reiter J., Zangen D. (2022). The utility of glucose area under the curve from the oral glucose tolerance test as a screening tool for cystic fibrosis-related diabetes. Pediatr. Pulmonol..

[56] Tong A., Wang D., Liu X., Li Z., Zhao R., Liu B., Zhao C. (2023). The Potential Hypoglycemic Competence of Low Molecular Weight Polysaccharides Obtained from *Laminaria japonica*. Foods.

[57] Diamond D.M., Alabdulgader A.A., de Lorgeril M., Harcombe Z., Kendrick M., Malhotra A., O’Neill B., Ravnskov U., Sultan S., Volek J.S. (2021). Dietary Recommendations for Familial Hypercholesterolaemia: An Evidence-Free Zone. BMJ Evid.-Based Med..

[58] Tsujita T. (2006). Basal lipolysis in epididymal fat cells from streptozotocin-induced diabetic rats. J. Nutr. Sci. Vitaminol..

[59] Chen Y., Yu L., Wang Y., Wei Y., Xu Y., He T., He R. (2019). D-Ribose contributes to the glycation of serum protein. Biochim. Biophys. Acta. Mol. Basis Dis..

[60] Xu F.X., Zhou X., Shen F., Pang R., Liu S.M. (2012). Decreased peripheral blood mitochondrial DNA content is related to HbA1c, fasting plasma glucose level and age of onset in type 2 diabetes mellitus. Diabet. Med. A J. Br. Diabet. Assoc..

[61] Song Q., Wu H., Weng S., Wang Y., Kong L., Liu Z., Zhang K. (2024). *Artemisia argyi* polysaccharide ameliorates hyperglycemia through modulating gut microbiota and serum metabolism in type 2 diabetic mice. J. Funct. Foods.

[62] Boer G.A., Holst J.J. (2020). Incretin Hormones and Type 2 Diabetes-Mechanistic Insights and Therapeutic Approaches. Biology.

[63] Richards P., Thornberry N.A., Pinto S. (2021). The gut-brain axis: Identifying new therapeutic approaches for type 2 diabetes, obesity, and related disorders. Mol. Metab..

[64] Yoshimura A., Yamaguchi T., Kugita M., Kumamoto K., Shiogama K., Ogitsu N., Yoneda M., Miura T., Nagamura Y., Nagao S. (2021). High Levels of Dietary Lard or Sucrose May Aggravate Lysosomal Renal Injury in Non-Obese, Streptozotocin-Injected CD-1 Mice Provided Isocaloric Diets. J. Nutr. Sci. Vitaminol..

[65] Nie Q., Hu J., Gao H., Li M., Sun Y., Chen H., Zuo S., Fang Q., Huang X., Yin J. (2021). Bioactive Dietary Fibers Selectively Promote Gut Microbiota to Exert Antidiabetic Effects. J. Agric. Food Chem..

[66] Zhang J.M., Sun Y.S., Zhao L.Q., Chen T.T., Fan M.N., Jiao H.C., Zhao J.P., Wang X.J., Li F.C., Li H.F. (2019). SCFAs-Induced GLP-1 Secretion Links the Regulation of Gut Microbiome on Hepatic Lipogenesis in Chickens. Front. Microbiol..

[67] Dalile B., Van Oudenhove L., Vervliet B., Verbeke K. (2019). The role of short-chain fatty acids in microbiota-gut-brain communication. Nat. Rev. Gastroenterol. Hepatol..

[68] Zhao Y., Wen J., Yang Y., Jia L., Ma Q., Jia W., Qi W. (2025). In vitro fermentation characteristics of polysaccharide from Scrophularia ningpoensis and its effects on type 2 diabetes mellitus gut microbiota. PeerJ.

[69] Yamashita H., Fujisawa K., Ito E., Idei S., Kawaguchi N., Kimoto M., Hiemori M., Tsuji H. (2007). Improvement of obesity and glucose tolerance by acetate in Type 2 diabetic Otsuka Long-Evans Tokushima Fatty (OLETF) rats. Biosci. Biotechnol. Biochem..

[70] Yao Y., Yan L., Chen H., Wu N., Wang W., Wang D. (2020). Cyclocarya paliurus polysaccharides alleviate type 2 diabetic symptoms by modulating gut microbiota and short-chain fatty acids. Phytomed. Int. J. Phyther. Phytopharm..

[71] Xiu W., Wang X., Na Z., Yu S., Wang J., Yang M., Ma Y. (2023). Ultrasound-assisted hydrogen peroxide-ascorbic acid method to degrade sweet corncob polysaccharides can help treat type 2 diabetes via multiple pathways in vivo. Ultrason. Sonochem..

[72] Han C., Kong X., Xia X., Huang X., Mao Z., Han J., Shi F., Liang Y., Wang A., Zhang F. (2023). Effects of ginseng peptides on the hypoglycemic activity and gut microbiota of a type 2 diabetes mellitus mice model. J. Funct. Foods.

[73] Hu B., Liu C., Jiang W., Zhu H., Zhang H., Qian H., Zhang W. (2021). Chronic in vitro fermentation and in vivo metabolism: Extracellular polysaccharides from Sporidiobolus pararoseus regulate the intestinal microbiome of humans and mice. Int. J. Biol. Macromol..

[74] Zhu Y., Chen B., Zhang X., Akbar M.T., Wu T., Zhang Y., Zhi L., Shen Q. (2024). Exploration of the Muribaculaceae Family in the Gut Microbiota: Diversity, Metabolism, and Function. Nutrients.

[75] Murakami A., Toyomoto K., Namai F., Sato T., Fujii T., Tochio T., Shimosato T. (2023). Oral administration of Brevibacterium linens from washed cheese increases the proportions of short-chain fatty acid-producing bacteria and lactobacilli in the gut microbiota of mice. Anim. Sci. J. Nihon Chikusan Gakkaiho.

[76] Wu R., Tang X., Kang X., Luo Y., Wang L., Li J., Wu X., Liu D. (2019). Effect of a Chinese medical nutrition therapy diet on gut microbiota and short chain fatty acids in the simulator of the human intestinal microbial ecosystem (SHIME). J. Funct. Foods.

[77] Chu S., Zhang F., Wang H., Xie L., Chen Z., Zeng W., Zhou Z., Hu F. (2022). Aqueous Extract of Guava (*Psidium guajava* L.) Leaf Ameliorates Hyperglycemia by Promoting Hepatic Glycogen Synthesis and Modulating Gut Microbiota. Front. Pharmacol..

[78] Holmberg S.M., Feeney R.H., Prasoodanan P.K.V., Puértolas-Balint F., Singh D.K., Wongkuna S., Zandbergen L., Hauner H., Brandl B., Nieminen A.I. (2024). The gut commensal Blautia maintains colonic mucus function under low-fiber consumption through secretion of short-chain fatty acids. Nat. Commun..

[79] Cani P.D., Knauf C. (2021). A newly identified protein from Akkermansia muciniphila stimulates GLP-1 secretion. Cell Metab..

[80] Yoon H.S., Cho C.H., Yun M.S., Jang S.J., You H.J., Kim J.H., Han D., Cha K.H., Moon S.H., Lee K. (2021). Akkermansia muciniphila secretes a glucagon-like peptide-1-inducing protein that improves glucose homeostasis and ameliorates metabolic disease in mice. Nat. Microbiol..

[81] Wan J., Yu X., Liu J., Li J., Ai T., Yin C., Liu H., Qin R. (2023). A special polysaccharide hydrogel coated on Brasenia schreberi: Preventive effects against ulcerative colitis via modulation of gut microbiota. Food Funct..

[82] Usta-Gorgun B., Yilmaz-Ersan L. (2020). Short-chain fatty acids production by Bifidobacterium species in the presence of salep. Electron. J. Biotechnol..

[83] Song W., Wang Y., Li G., Xue S., Zhang G., Dang Y., Wang H. (2023). Modulating the gut microbiota is involved in the effect of low-molecular-weight Glycyrrhiza polysaccharide on immune function. Gut Microbes.

[84] Sanmiguel C.P., Jacobs J., Gupta A., Ju T., Stains J., Coveleskie K., Lagishetty V., Balioukova A., Chen Y., Dutson E. (2017). Surgically Induced Changes in Gut Microbiome and Hedonic Eating as Related to Weight Loss: Preliminary Findings in Obese Women Undergoing Bariatric Surgery. Psychosom. Med..

[85] Pan X., Meng J., Xu L., Chang M., Feng C., Geng X., Cheng Y., Guo D., Liu R., Wang Z. (2022). In-depth investigation of the hypoglycemic mechanism of Morchella importuna polysaccharide via metabonomics combined with 16S rRNA sequencing. Int. J. Biol. Macromol..

[86] Ni Z., Chen L., Qian X., Yong Y., Wu M., Li Y., Li J., Wang Y., Li L., Shao Y. (2025). Preliminary characterization of Ramaria botrytoides polysaccharide RB-P1-1 and analysis of its hypoglycemic effects by altering the gut microbiota and metabolites in mice with type 2 diabetes mellitus. Int. J. Biol. Macromol..

[87] Li S., Chen W., Ma S., Zhou X., Li J., Li B. (2025). Expandable konjac fiber modulates appetite and chyme digestion in vivo by stomach-intestine-brain axis. Int. J. Biol. Macromol..

[88] Darsalia V., Nathanson D., Nyström T., Klein T., Sjöholm Å., Patrone C. (2014). GLP-1R activation for the treatment of stroke: Updating and future perspectives. Rev. Endocr. Metab. Disord..

[89] Li L., Wei T., Liu S., Wang C., Zhao M., Feng Y., Ma L., Lu Y., Fu P., Liu J. (2021). Complement C5 activation promotes type 2 diabetic kidney disease via activating STAT3 pathway and disrupting the gut-kidney axis. J. Cell. Mol. Med..

[90] Turton M.D., O’Shea D., Gunn I., Beak S.A., Edwards C.M., Meeran K., Choi S.J., Taylor G.M., Heath M.M., Lambert P.D. (1996). A role for glucagon-like peptide-1 in the central regulation of feeding. Nature.

[91] Cao S., Liu M., Han Y., Li S., Zhu X., Li D., Shi Y., Liu B. (2024). Effects of Saponins on Lipid Metabolism: The Gut-Liver Axis Plays a Key Role. Nutrients.

