# Supplementary material for: Structural Characterization and Hypoglycemic Mechanism of Pumpkin Seed Polysaccharides
Source: Foods. 2026 Oct 22;15(21):3788. doi: 10.3390/foods15213788 (PMC13763181; doi:10.3390/foods15213788)
Supplement: Supplementary file 1 [file foods-15-03788-s001.zip › foods-4547244-supplementary.pdf]

Supplementary material

Table S1. The physicochemical properties and yields of each pumpkin seed polysaccharide fraction.

Components	Neutral sugar	Uronic acid	Protein	Yield
PSP-1	43.46±0.27	15.27±0.41	18.4±0.27	23.22±0.66
PSP-1-1	92.15±1.55	9.76±0.76	5.10±0.74	8.34±0.54
PSP-1-2	81.31±2.61	4.09±0.16	8.82±1.32	20.33±0.34
PSP-1-3	85.87±1.73	2.57±0.16	10.78±1.80	24.00±0.45
PSP-1s	84.05±1.33	4.24±0.06	9.01±1.15	53.33±0.83

The yields of PSP-1 mentioned above are based on the loading amount onto the DEAE-52 column, while the yields of PSP-1-1, PSP-1-2, PSP-1-3, and PSP-1s are based on the loading amount onto the Experdex-75 column.

Table S2. RT-qPCR primer design for determination of sweet taste receptor signaling pathway-related gene expression in NCI-H716 cells.

Genes	Forward (5'→3')	Reverse (5'→3')
T1R2	TCCCTATGTCCATGTGTTCCAA	TGCCCACAGGCTTCTTCTTT
T1R3	CGTGAGCGCAGGGCTAAA	ACGCTATACACAGCTGCGTAGA
Gnat3	GTGAAAGCCATGACTACCCTTGG	CTCAGCCAGTTGAGGTGTCATG
PLCβ2	CCTGGAAGTGACGGCTTATGAG	GCTCTGTGAAGGACGAGATGAC
IP3R	GCAACCACATCTGGACGCTCTT	AGAAGGCGTTGATGGTGTCCAG
TRPM5	GCGTACTCAAGGACTTCCTGCA	TTCAGGTCCAGCAGCCACTTCT
GLUT2	ATGTCAGTGGGACTTGTGTGTGC TGC	AACTCACCCACCATGAACCAGG
SGLT1	CGCCTATCCAACCTTAGTGGTG	CGCTGTTGAAGATGGAGGTCAG
β-actin	CCTTCCTGGGCATGGAGTCCTG	GGAGCAATGATCTTGATCTTC

Table S3. SCFAs reference standards calibration curve.

SCFAs	Calibration curve	R^2
Acetic acid	y=8810.86x+261599.60	0.9993
Propionic acid	y=6354.29x-23600.69	0.9996
Butyric acid	y=12961.17x-9.13	0.9997
Isobutyric acid	y=12863.99x-3988.28	0.9990
Valeric acid	y=15254.92x-41119.06	0.9993
Isovaleric acid	y=15354.22x-22453.56	0.9995

Table S4. RT-qPCR primer design for determination of appetite-related gene expression in mice hypothalamus.

Genes	Forward (5'→3')	Reverse (5'→3')
POMC	TAGATGTGTGGAGCTGGTGC	CGTACTTCCGGGGGTTTTCA
CART	AAGAAGTACGGCCAAGTCCC	CAGTCACACAGCTTCCCGAT
NPY	TGTGTTTGGGCATTCTGGCTGAG	TGTAGTGTCGCAGAGCGGAGTAG
AgRP	GCTTTGGCGGAGGTGCTAGATC	AAGGCATTGAAGAAGCGGCAGTAG
β-actin	AACAGTCCGCCTAGAAGCAC	CGTTGACATCCGTAAAGACC

Table S5. Glycosyl linkages analysis of PSP-1-1.

Type of linkage	Mass fragments (m/z)	Retention time (min)	Relative molar ratio (%)
Ara(f)-(1→	43,71,87,102,118,129,145,161	5.848	20.82
Gal(p)-(1→	43,87,102,118,129,145,161,162,205	8.646	13.04
Glc(p)-(1→	43,87,102,118,129,145,161,162,205	9.622	5.56
→5)-Ara(f)-(1→	43,45,71,87,99,101,117,129,161,189	10.369	9.93
→2,3)-Rha(p)-(1→	43,71,89,101,131,262	11.528	1.56
→3)-Gal(p)-(1→	43,87,101,118,129,161,202,234	12.464	10.70
→4)-Gal(p)-(1→	43,87,102,113,118,129,162,233	13.738	8.75

→6)-Gal(p)-(1→	43,87,99,102,118,129,162,189,233	15.109	3.75
→4,6)-Gal(p)-(1→	43,85,102,118,127,159,162,201,261	17.966	2.61
→3,6)-Gal(p)-(1→	43,87,101,118,129,160,189,234	18.508	20.85
→3,4,6)-Gal(p)-(1→	43,97,118,129,139,160,333	19.968	2.43

Table S6. Glycosyl linkages analysis of PSP-1-2.

Type of linkage	Mass fragments (m/z)	Retention time (min)	Relative molar ratio (%)
Ara(f)-(1→	43,71,87,102,118,129,145,161	5.848	8.22
Xyl(p)-(1→	43,71,87,102,118,161	7.145	7.69
Gal(p)-(1→	43,87,102,118,129,145,161,162,205	8.646	1.97
Glc(p)-(1→	43,87,102,118,129,145,161,162,205	9.622	14.63
→5)-Ara(f)-(1→	43,45,71,87,99,101,117,129,161,189	10.369	8.65
→3)-Gal(p)-(1→	43,87,101,118,129,161,202,234	12.464	8.07
→4)-Glc(p)-(1→	43,87,99,101,113,117,129,131,161,173,233	13.630	15.20
→4)-Gal(p)-(1→	43,87,102,113,118,129,162,233	13.738	3.18
→6)-Gal(p)-(1→	43,87,99,102,118,129,162,189,233	15.109	2.09
→3,4)-Glc(p)-(1→	43,87,98,118,143,185,231,305	15.787	1.92
→2,4)-Glc(p)-(1→	43,87,113,130,173,190,214,233,274	16.433	1.77
→4,6)-Glc(p)-(1→	43,85,87,99,101,117,127,142,159,187,201,261	17.855	1.36
→4,6)-Gal(p)-(1→	43,85,102,118,127,159,162,201,261	17.966	1.94
→3,6)-Galp-(1→	43,87,101,118,129,160,189,234	18.508	23.31

Table S7. Glycosyl linkages analysis of PSP-1-3.

Type of linkage	Mass fragments (m/z)	Retention time (min)	Relative molar ratio (%)
Ara(f)-(1→	43,71,87,102,118,129,145,161	5.848	7.38
Xyl(p)-(1→	43,71,87,102,118,161	7.145	11.57
Gal(p)-(1→	43,87,102,118,129,145,161,162,205	8.646	4.70
Glc(p)-(1→	43,87,102,118,129,145,161,162,205	9.622	8.21
→5)-Ara(f)-(1→	43,45,71,87,99,101,117,129,161,189	10.369	8.80
→3)-Gal(p)-(1→	43,87,101,118,129,161,202,234	12.464	16.78
→4)-Glc(p)-(1→	43,87,99,101,113,117,129,131,161,173,233	13.630	2.86
→4)-Gal(p)-(1→	43,87,102,113,118,129,162,233	13.738	3.69
→6)-Gal(p)-(1→	43,87,99,102,118,129,162,189,233	15.109	4.94
→3,4)-Glc(p)-(1→	43,87,98,118,143,185,231,305	15.787	1.97
→2,4)-Glc(p)-(1→	43,87,113,130,173,190,214,233,274	16.433	1.52
→4,6)-Glc(p)-(1→	43,85,87,99,101,117,127,142,159,187,201,261	17.855	19.33
→4,6)-Gal(p)-(1→	43,85,102,118,127,159,162,201,261	17.966	3.79
→3,6)-Gal(p)-(1→	43,87,101,118,129,160,189,234	18.508	4.46

Table S8. α-diversity analysis of the gut microbiota in diabetic mice.

α-diversity	Con	Model	H-PSP-1s
Goods Coverage	0.99996 ± 0.0003^{ab}	0.99996 ± 0.00003^{a}	0.99992 ± 0.00004^{b}
Chao1	217.39 ± 29.08^{a}	169.41 ± 20.11^{b}	219.52 ± 30.22^{a}
Observed Species	217.13 ± 29.13^{a}	169.20 ± 20.12^{b}	218.57 ± 29.75^{a}
PD whole tree	17.00 ± 1.47^{a}	14.13 ± 1.03^{b}	16.77 ± 1.20^{a}

Different letters represent significant differences in all groups ($P<0.05$). n=6.

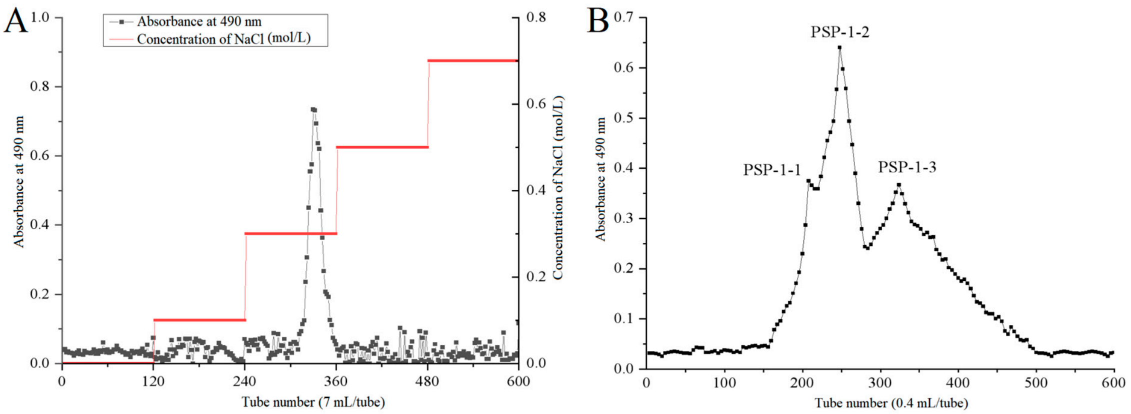

Figure S1. Isolation and Purification of Pumpkin Seed Polysaccharides: (A) DEAE-52 cellulose column elution curve and (B) Sephadex-75 gel column elution curve.
